# Nuclear Localization of BRAF^V600E^ Is Associated with HMOX-1 Upregulation and Aggressive Behavior of Melanoma Cells

**DOI:** 10.3390/cancers14020311

**Published:** 2022-01-09

**Authors:** Mourad Zerfaoui, Eman Toraih, Emmanuelle Ruiz, Youssef Errami, Abdallah S. Attia, Moroz Krzysztof, Zakaria Y. Abd Elmageed, Emad Kandil

**Affiliations:** 1 Department of Surgery, Tulane University School of Medicine, New Orleans, LA 70112, USA; etoraih@tulane.edu (E.T.); remmanuelle@tulane.edu (E.R.); yerrami@tulane.edu (Y.E.); aattia@tulane.edu (A.S.A.); zelmageed@ulm.vcom.edu (Z.Y.A.E.); ekandil@tulane.edu (E.K.); 2 Department of Pathology, Tulane University School of Medicine, New Orleans, LA 70112, USA; kmoroz@tulane.edu; 3 Department of Pharmacology, Edward Via College of Osteopathic Medicine, University of Louisiana, Monroe, LA 71203, USA

**Keywords:** melanoma, HMOX-1, nuclear BRAF^V600E^, aggressiveness, vemurafenib resistance, poor prognosis

## Abstract

**Simple Summary:**

Despite some successes of selective anti-BRAF^V600E^ inhibitors, resistance remains a major challenge. The aim of our study is to determine the role of nuclear BRAF^V600E^ and its newly identified partner, HMOX1, in melanoma aggressiveness and drug resistance. We identified the mechanism by which drug resistance is developed via the nuclear localization of BRAF^V600E^ and its partner HMOX1 in melanoma tissues and cell lines. According to our studies, the outcomes of our manuscript have a direct clinical impact on establishing novel prognostic markers and therapeutic intervention strategies in metastatic melanoma. This study provides new information on the ability to selectively classify patients with cytosolic BRAF for selective BRAF inhibitors and offers an alternative treatment to patients with nuclear BRAF^V600E^ and high HMOX1 expressions.

**Abstract:**

Background: Previously, we have demonstrated that nuclear BRAF^V600E^ is associated with melanoma aggressiveness and vemurafenib resistance. However, the underlying mechanisms of how nuclear localization of BRAF^V600E^ promotes cell aggressiveness have not yet been investigated. Despite therapeutic advancements targeting cutaneous melanoma, unknown cellular processes prevent effective treatment for this malignancy, prompting an urgent need to identify new biological targets. This study aims to explore the association of inducible heme oxygenase 1 (HMOX-1) with nuclear BRAF^V600E^ in promoting melanoma aggressiveness. Methods: Proteomics analysis was performed to identify the interacting partner(s) of nuclear BRAF^V600E^. Immunohistochemistry was applied to evaluate the levels of HMOX-1 and nuclear BRAF^V600E^ expression in melanoma and adjacent healthy tissues. Immunofluorescence assessed the nuclear localization of BRAF^V600E^ in vemurafenib-resistant A375R melanoma cells. Further study of HMOX-1 knockdown or BRAF^V600E^ overexpression in melanoma cells suggested a role for HMOX-1 in the regulation of cell proliferation in vivo and in vitro. Finally, Western blot analysis was performed to confirm the pathway by which HMOX-1 mediates Akt signaling. Results: Proteomics results showed that HMOX-1 protein expression was 10-fold higher in resistant A375R cells compared to parental counterpart cells. In vitro and in vivo results illustrate that nuclear BRAF^V600E^ promotes HMOX-1 overexpression, whereas HMOX-1 reduction represses melanoma cell proliferation and tumor growth. Mechanistic studies revealed that HMOX-1 was associated with nuclear BRAF^V600E^ localization, thus promoting melanoma proliferation via a persistent activation of the AKT pathway. Conclusions: Our results highlight a previously unknown mechanism in which the nuclear BRAF^V600E^/HMOX-1/AKT axis plays an essential role in melanoma cell proliferation. Targeting HMOX-1 could be a novel method for treating melanoma patients who develop BRAF inhibitor resistance.

## 1. Introduction

Melanoma is an aggressive solid neoplasm with a highly critical prognosis. Surgery and combinational chemotherapies have been shown to confer only modest survival benefits in advanced melanoma, with a five-year survival rate below 16% in patients with metastatic melanoma [1]. Recently, an expanded understanding of the molecular mechanisms of melanoma genesis has reformed its treatment. Approximately 50% of cutaneous melanomas harbor *BRAF* mutations, which are mainly due to a point mutation—glutamic acid for valine—at codon 600 (*BRAF^V600E^*) [2]. *BRAF* mutations lead to the constitutive activation of ERK in the MAPK signaling pathway and provide cells with the ability to evade apoptosis, increase invasiveness, and escape from the immune system [3]. Although there are many unanswered questions related to advanced-stage *BRAF^V600E^* melanomas, the major indecision in current practice is the choice between BRAF/MEK inhibitors or immunotherapy for metastatic or high-risk disease patients [4]. For the BRAF inhibitors, novel oncogene-targeted therapies have been developed; for instance, vemurafenib/PLX4032 has proved to be very effective in BRAF^V600E^-positive metastatic melanoma by favoring tumor regression [5]. Unfortunately, many patients show a relapse of the disease in less than 12 months [6]. Reactivation of the MAPK pathway is the major cause for the development of drug resistance to BRAF inhibitors. Although BRAF inhibitors are efficient in decreasing cell proliferation via the inhibition of the MAPK/ERK pathway, reactivation of this pathway occurs in 80% of BRAF-inhibitor-resistant cancer cells, suggesting that these cells rapidly adapt to MAPK inhibition [7]. Furthermore, melanoma cells can adapt their metabolic activities to deal with reactive oxygen species (ROS)-induced damage. NRF2 (nuclear factor (erythroid-derived 2)-like 2) is a transcription factor that regulates the antioxidative response as a result of ROS and protects cells against oxidative damage. This metabolic adaptation allows BRAF inhibitor-resistant melanoma cells to survive under oxidative stress [8]. Heme oxygenase 1 (HMOX-1) has the most abundant antioxidant response element (ARE) in the promotion of genes regulated by *Nrf2* and has been reported to be very important in preventing diseases triggered by oxidative stress [9,10].

HMOX-1 is an inducible enzyme that can provide a strong cytoprotective response against a wide range of stressors, including oxidative stress, radiation, heavy metals, and inflammatory cytokines. It carries out the degradation of heme groups and generates biliverdin, which is further converted into bilirubin by biliverdin reductase (BVR), carbon monoxide (CO), and free iron. This effect is mediated, at least in part, via the activation of the PI3K/Akt pathway [11]. Overall, HMOX-1 metabolic products exert antioxidant, anti-inflammatory, and antiapoptotic activities [12].

HMOX-1 induction has been highlighted as a mechanism involved in cancer progression, playing a role in cancer growth, metastatic potential, invasiveness, angiogenesis, and resistance to therapies in many cancers, including multiple myeloma [13], acute leukemia [14], and neuroblastoma [15,16,17]. Moreover, a previous study showed that high expression of HMOX-1 was associated with tumor aggressiveness and BRAF^V600E^ expression in a subset of thyroid cancers [18]. A recent study highlighted the important role of NRF2 and HMOX-1 upregulation in the chemoresistance of KRAS-mutant pancreatic cancers [19].

Some cancer cells use the cellular processes of nuclear-cytoplasmic transport to effectively develop resistance to established therapies [20,21,22]. In such cases, a number of tumor suppressor proteins change their subcellular localization, dysregulating their tumor-suppressor activities. Many of these proteins are transported from the nucleus to the cytosol by the action of the exporting protein 1, CRM1 [22]. A continuous but not transient ERK activation is connected to its nuclear localization, suggesting that the differential activation of ERK may be a determinant factor for eliciting a specific biologic effect [23]. Therefore, the cellular compartments of RAS/RAF/MEK/ERK are very important for exerting a certain biologic role during normal and pathologic conditions. In our recent study, we found a clear role of the BRAF^V600E^ nuclear localization in melanoma aggressiveness and drug resistance [24]; however, little is known about the mechanisms that govern this new phenomenon.

In the current study, we demonstrate for the first time that BRAF^V600E^ nuclear localization upregulates 17 cancer-related proteins, with HMOX-1 as the most upregulated. We also report the association of BRAF^V600E^ translocation to the nucleus and high protein expression of HMOX-1. The depletion of HMOX-1 using specific shRNA lentivirus particles inhibited cell and tumor proliferation in vitro and in vivo. Moreover, bioinformatic analysis showed higher HMOX-1 expression levels in resistant mice inoculated with A375 cells and treated with BRAF inhibitors compared to sensitive animals. Additional bioinformatic analysis for sensitive and resistant patients after BRAF inhibitor treatment showed that HMOX-1 was upregulated in resistant patients.

## 2. Materials and Methods

### 2.1. Cell Culture, Treatment, and Lentiviral Infection

All cells used in the study (A375, A375R, WM983B, SKMEL-28, MEF-/-BRAF, and MV3) were cultured as previously described [24]. Briefly, cells were cultured in DMEM medium (American Type Culture Collection, ATCC, Manassas, VA, USA) containing 10% fetal bovine serum, 0.1 mM nonessential amino acids, 1 mM sodium pyruvate, and 1% penicillin–streptomycin and maintained at 37 °C in a humidified 5% CO_2_ incubator. The medium was replaced every two days with a fresh medium to maintain cell activity. We routinely use the PlasmoTest™—Mycoplasma Detection Kit (Cat.code: rep-pt1) from Invivogen to detect mycoplasma. We use the cells only if they are negative to the mycoplasma test. The A375 melanoma cells (V600E mutant) and their resistant A375R cells were kindly provided by Dr. Andrew E. Aplin (Thomas Jefferson University, Philadelphia, PA, USA). MV3 (*BRAF* wild-type) cells were provided by Dr van Muijen (Radboud University Medical Center, Nijmegen, The Netherlands). MEF (*BRAF* KO) cells were kindly provided by Dr. Manuela Baccarini (Austria). For cellular translocation, A375 and A375R were seeded in a chamber slide without FBS for synchronization. After overnight culture, the cells were exposed to DMEM containing 10% FBS for 1, 3, 6, and 9 h, and the cells were subjected to immunofluorescence with specific antibodies. For lentiviral infection, HMOX1 shRNA particles (sc-35554-V) and control shRNA lentiviral particles (sc-108080) were transduced in the presence of polybrene (5 μg/mL) into A375R cells with a multiplicity of infection (MOI) of 1 or 10 overnight. After changing the media and incubating the cells for an extra 48 h, puromycin dihydrochloride (sc-108071) is used to select stable clones. 

### 2.2. Immunohistochemistry (IHC) 

To assess HMOX-1 and BRAF^V600E^ cellular localization of their protein expression within tumor tissue, we performed IHC on formalin-fixed paraffin-embedded human and animal specimens, as previously described [25]. Briefly, 4-mm thick tissue slides were subjected to deparaffinization in xylene, then rehydrated in serial dilutions of alcohol and immunostained with Biocare reagents on a Biocare Nemesis 7200 automated system (Biocare Medical, Pacheco, CA, USA). Endogenous biotin and peroxidase activity were quenched with sequential incubation of tissue slides in 3% hydrogen peroxide for 5 min and in an avidin–biotin blocking solution for 10 min. Borg Declocker RTU (Biocare Medical) was used for antigen retrieval to prevent nonspecific binding, and tissue was then blocked with Background Sniper solution (Biocare Medical). Immunostaining was performed using an anti-human BRAF^V600E^ monoclonal VE1 antibody (#E19290, Spring Bioscience, Pleasanton, CA, USA) and an anti-HMOX-1 antibody (sc-136960 A-3, Santa Cruz Biotechnology, Dallas, TX, USA) at a 1:100 dilution for 60 min and subsequently incubated with a horseradish peroxidase-conjugated secondary antibody for 25 min. The immunostaining signal was developed using diaminobenzidine substrate-chromogen solution. The tissue slides were counterstained with hematoxylin and bluing solution. Negative controls were processed by replacing the antibody with phosphate-buffered saline (PBS). Staining intensity was scored in a blinded manner on a scale of 0 to 3, with 0 representing no staining, 1 low staining, 2 moderate staining, and 3 high staining. Localization of HMOX-1 and BRAF^V600E^ was recorded as cytoplasmic and/or nuclear staining per tissue section. A human malignant melanoma tissue array was purchased from Biomax (ME551).

### 2.3. Cell Proliferation

Cells were seeded overnight into 96-well plates. The next day, they were treated with vemurafenib (PLX4032, SellekChem) at different concentrations (0 to 5 mM). After 3 days of treatment, cell viability was assessed using 3-(4,5-dimethyl-thiazol2-yl)-2,5-diphenyltetrazolium bromide (MTT, Sigma-Aldrich, St. Louis, MO, USA). The cells were incubated with MTT working solution (10% of MTT 5 mg/mL) for 2 h at 37 °C, and then the insoluble formazan salts were dissolved in DMSO. The absorbance at 570 nm was measured with a spectrophotometric plate reader. Mean values from each treatment were calculated as a percentage relative to the untreated cells. For each cell line, the assay was performed in triplicate, and each experiment was independently repeated at least three times.

### 2.4. Clonogenic Assay

Briefly, 500 cells of A375, A375-NLS-BRAF^V600E^, A375R, and A375R-HMOX1 KD were seeded in a 100 mm plate with 10 mL of complete medium for 14 days. At the end of 14 days, the medium was aspirated from each well and a mixture of 50% methanol and 1% methylene blue was added. The cells were incubated in this solution for 30 min for fixation and staining. After the plates had dried out, the colony number per well was counted at least in triplicate. 

### 2.5. Wound Healing and Invasion Assay

For wound healing, the melanoma cells (A375R, A375R-CTR LV, A375R-HMOX1 KD) were seeded onto 6-well plates, grown to 100% confluence, wounded with a sterile pipette tip, and treated or not with an antimitotic agent: Vindoline. Wound closure was photographed immediately following the wound and 16 h later. The percentage of wound healing was reported as the healed area width divided by the total wounded area width. The assay was performed in triplicate, and each experiment was independently repeated at least 3 times.

For the effect of HMOX1 KD on melanoma cell migration, 1 × 10^6^ and/or 2 × 10^6^ A375R and WM983B were cultured in the upper chamber of the NUNC (Thermo Scientific (140663), Waltham, MA, USA). The lower wells contained growth media with 10% FBS. The chambers were incubated for 24 h at 37 °C. After the removal of the non-migrated cells on top of the filter, the cells that had migrated through the membrane were fixed in and then stained with a Quick III stain set (UN 1230) from Astraldiagnostics. The number of cells that had migrated into the lower chamber was counted in 6 randomly selected high-power microscopic fields.

### 2.6. Immunofluorescence Analysis

Cells were cultured in chamber slides under regular and experimental conditions. At the end of each experiment, the cells were fixed with 4% paraformaldehyde (Wako Laboratory Chemicals) at room temperature. After washing, cell membranes were permeabilized by treating the cells with 0.1% Triton X for 15 min and then incubating the cells with 5% FBS blocking solution for 1 h. The cells were incubated overnight at 4 °C in a primary antibody (VE1 clone; Spring Bioscience or HMOX-1 A-3, Santa Cruz). The cells were incubated with Alexa Fluor 594 or 488 secondary antibodies. This step was followed by staining cells with 4′, 6-diamindino-2-phenylindole (DAPI) and adding mounting medium (Vector Laboratories, Burlingame, CA, USA). The developed color was acquired by an Olympus BX51 fluorescence microscope (Olympus America Inc., Center Valley, PA, USA).

### 2.7. Western Blot Analysis

Cells were lysed in RIPA lysis buffer, including protease inhibitors, according to the manufacturer’s instructions (Santa Cruz Biotechnology, Dallas, TX, USA). Protein concentrations were determined using the Bradford method (Bio-Rad). A total of 20 μg protein lysates were separated on 4–12% SDS-polyacrylamide gels and transferred onto polyvinylidene fluoride membranes (Bio-Rad). For detection of BRAF^V600E^, HMOX-1, phosphorylated AKT (B-12 sc-377556), phosphoERK (Cell Signaling #4370), GAPDH (G-9 sc-365062), and actin (C-2: sc-8432)-specific antibodies were used at 1:1000 dilution, and protein signals were developed by an ECL advance detection kit according to the manufacturer’s instructions (Amersham Bioscience, Piscataway, NJ, USA). Uncropped Western Blot images could be found at Original blots and intensity ratio.

### 2.8. Generation of Clones Stably Expressing the Nuclear Localization Signal of BRAF^V600E^

MEF (*BRAF* KO) and MV3 (*BRAF* wild-type) melanoma cells expressing nuclear localization signal (NLS)-BRAF^V600E^ and BRAF^V600E^ were established by infecting the cells with lentiviral particles expressing human BRAF^V600E^ with NLS sequence pLV[Exp]-Neo-CMV>DsRed_Express2: ORF_2373bp/ Myc(hBRAF^V600E^/3xNLS) or without NLS pLV[Exp]-NeoCMV>DsRed_ Express2:ORF_2301bp/Myc(hBRAF^V600E^) tagged with DsRed fluorescent protein according to the standard protocols (Cyagen, Santa Clara, CA, USA). After two weeks of 0.25 mg/mL G418 selection, the growing colonies were examined for expression of NLS-BRAF^V600E^ and BRAF^V600E^ using immunofluorescence and Western blot analyses. The MV3 cells were generated for the in vivo study. The MEF cells were generated for the in vitro study.

### 2.9. Xenograft Melanoma Model

All animal experiments were performed in accordance with the ethical guidelines of the Institutional Animal Care and Use Committee of Tulane University, New Orleans, LA, USA. For the analysis of in vivo tumor growth and invasion, 3 × 10^6^ of A375R and A375R/HMOX-1 KD cells in 200 μL of DMEM were subcutaneously injected into each 4- to 6-week-old nude mouse (The Jackson Laboratory, Bar Harbor, ME, USA) (n = 9 for A375R and n = 11 for A375R/HMOX-1 KD). Calipers were used to measure the tumor size every 3 days. At 25 days after injection, the mice were euthanized, and the tumors were surgically isolated, weighed, and preserved for further analyses.

For the MV3 experiment, nude female mice (Charles River, Wilmington, MA, USA), about 6 weeks old, were housed in a pathogen-free barrier facility. The mice (5 mice per group) were subcutaneously inoculated with 0.5 × 10^6^ MV3, MV3-BRAF^V600E^ or MV3- NLS-BRAF^V600E^ cells with an equal amount of Matrigel basement membrane matrix (BD Bioscience) in the right flank, and tumor growth was monitored twice a week with calipers. At the end of the experiment, mice were sacrificed by euthanasia, and the tumor tissues were harvested for immunohistochemistry with HMOX-1 and BRAF^V600E^ antibodies.

### 2.10. Bioinformatics Analysis

The cancer genome atlas (TCGA) melanoma cohort RNA-seq gene expression was downloaded from the Firebrowse platform (http://firebrowse.org/, accessed on 6 September 2021). *BRAF* mutation and the survival and clinical information of 406 patients corresponding to the RNA-seq data were collected from the cbioportal platform (https://www.cbioportal.org/, accessed on 6 September 2021). The expression of the *HMOX-1* gene was compared with different clinical and survival data. Mann–Whitney U and Kaplan–Meier survival curves were performed to assess the association of HMOX-1 expression with clinical parameters. Survival analyses were run through Kaplan–Meier curve analysis and the Cox regression model to estimate disease-free survival (DFS). Meier survival curves were plotted using the *survival* R package, and the log-rank test was used for significance. The optimized cut-off between “high” and “low” HMOX1 expression was identified with the *survminer* R package.

The A375 xenograft samples were retrieved from the GSE74729 GEO dataset (https://www.ncbi.nlm.nih.gov/gds, accessed on 6 September 2021). A differential expressed gene analysis was performed to compare vemurafenib-resistant A375 cell line tumors (5 A375-R samples) with vemurafenib-sensitive tumors (4 A375 samples) with the *DEGseq* R package [26]. The melanoma patient cohort (43 BRAF inhibitors—sensitive before treatment, and 48—resistant after treatment and relapse of disease) comprised 3 public cohorts (GSE99898, GSE50509, GSE77940). *HMOX-1* expression was normalized across the databases with the z-score method. Z-score values were used for the statistical analysis. Statistical analyses were performed with the Mann–Whitney U-test (*kruskal.test)* R function. 

KEGG pathways analyses were performed on the two databases independently with the *pathfinder* R package using the active subnetwork-oriented pathway enrichment method. Enrichment scores were plotted with the *ggplot2* R package. A heatmap of the correlation matrix was performed after a hierarchical clustering and optimization of the number of clusters cut-off based on the silhouette index. *clValid, factoextra,* and *pheatmap* R packages were used. Finally, the network was designed with Cytoscape 3.8 software using Spearman’s correlation values.

### 2.11. Apoptosis Quantification

To determine apoptosis, the phosphatidylserine sensor Apopxin was used. CytoCalcein Violet 450 was used to mark live cells. All reagents were purchased in kit form from Abcam (ab176749). After HMOX-1 knockdown or vemurafenib treatment or control conditions, A375R cells were incubated with two sensors simultaneously (one for live cells and one for apoptosis) for 30 min at room temperature. The cells were then washed twice and imaged using fluorescence microscopy.

### 2.12. Statistical Analysis

All values are expressed as the mean ± SD. Statistical analysis of the data was performed by a two-tailed Student’s *t*-test, and significance was considered at *p* < 0.05.

## 3. Results

### 3.1. Translocation of BRAF^V600E^ into the Nucleus Upregulates HMOX-1 Expression in Melanoma Cell Lines

**Figure 1:**
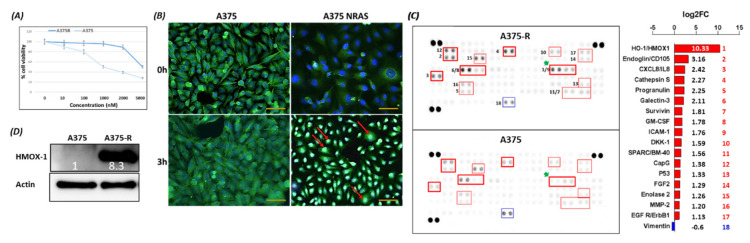
Protein expression of HMOX-1 in parental (P) and resistant A375 (R) cell lines. (**A**) MTT assay was assessed on parental and resistant A375 cell lines after treatment with different concentrations of vemurafenib for 3 days. This experiment was performed in triplicate and independently repeated three times. (**B**) IF with BRAF^V600E^ antibody. Cells were synchronized and then stimulated with FBS for 3 h. Scale bar = 20 μm (**C**) Human XL oncology array (relative levels of 84 human cancer-related proteins) incubated with total protein extractions collected from parental and resistant A375 cell lines. (**D**) HMOX-1 protein levels in parental A375 cells and resistant A375 cells.

To determine the differential response of melanoma cells towards PLX-4032 treatment, two *BRAF^V600E^* melanoma cell lines, parental and resistant (A375P and A375R) cells, were subjected to 10, 100, 1000, 2000, and 5000 ng of PLX-4032 treatment for 3 days. MTT assay revealed that cell viability starts to decline in A375P at 10 and 100 ng and is drastically reduced at the range of 1–5 μM of the drug. However, A375R showed a substantial resistance up to 2 μM of vemurafenib (Figure 1A). This experiment clearly shows that A375R cells are resistant to vemurafenib treatment. In a previous study, we demonstrated that the conventional nuclear BRAF^V600E^ localization was associated with melanoma aggressiveness and vemurafenib resistance [24]. In order to explore whether BRAF^V600E^ has a nuclear localization in A375R cells, we monitored the cellular localization after 24 h of cell synchronization followed by serum stimulation. The A375R cells presented a pronounced nuclear translocation of the mutant kinase compared to the parental cells after only 3 h of serum stimulation (Figure 1B), confirming a similar finding with a SkMel-28 cell line stimulated with serum or epidermal growth factor (EGF) in our previous study [24]. BRAF^V600E^ nuclear localization was validated by cell fractionation of the SkMel-28 cells (Figure S8). Taken together, these results suggest that nuclear BRAF^V600E^ localization could be a determinant factor in the resistance to vemurafenib of A375R cells. In our effort to identify the partner(s) of nuclear BRAF^V600E^ in resistant melanoma cells, we took a proteomics approach. We examined the differential expressed proteins in A375P and A375R cells using the Human XL Oncology Array, as recommended by the manufacturer’s protocol (Cat# ARY026; R&D Systems, USA). The full list of the 84 cancer-related proteins of the array is provided in the supplementary material (Figure S4). The screening of the protein lysates of resistant and parental cells identified one downregulated and 17 upregulated specific melanoma-related proteins. Among the 17 upregulated proteins, HMOX-1, a partner to nuclear BRAF^V600E^, was the most upregulated (10.3-fold) protein (Figure 1C). The high expression level of the HMOX-1 protein in A375R cells compared to A375P cells was confirmed by Western blot analysis (Figure 1D), PCR, and bioinformatic analysis (Figure S9A,B). These data suggest that HMOX-1 may be the most prominent nuclear BRAF^V600E^ partner to reduce the efficiency of vemurafenib treatment and promote cell proliferation.

### 3.2. HMOX-1 Gene Expression Is Associated with an Aggressive Phenotype, a Worse Prognosis, and Resistance to BRAF Inhibitor

*HMOX-1* association with aggressive features and survival was studied in 406 TCGA melanoma samples. A higher expression of *HMOX1* was correlated with more aggressive lymph node stages (N2 and N3, more than 2 lymph nodes invaded) compared to the absence of lymph node metastasis (N0) (*p* = 0.005).

**Figure 2:**
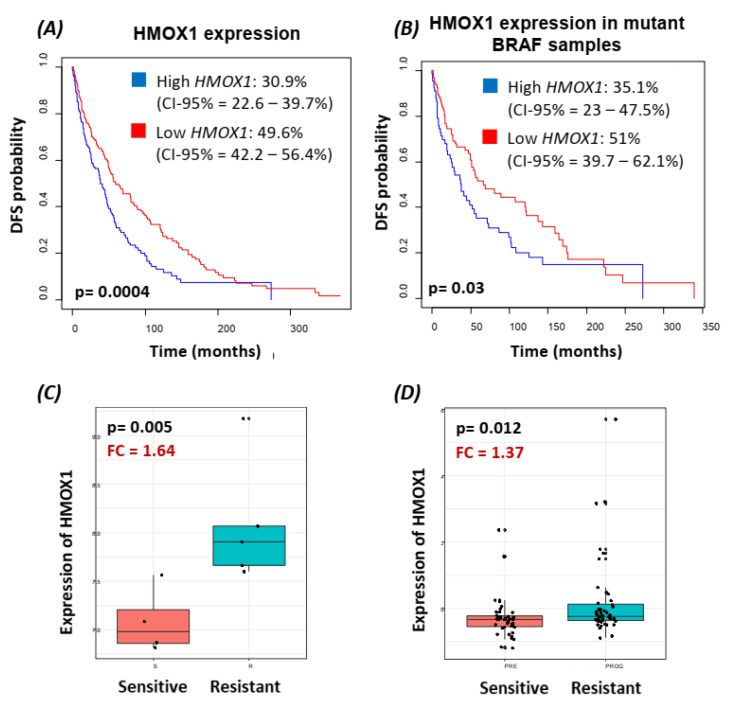
Transcriptomic analysis on public datasets highlighted the role of HMOX-1 in aggressiveness and BRAF inhibitor resistance in melanoma. (**A**,**B**) Kaplan–Meier curves representing the disease-free survival (DFS) of patients with a high expression of HMOX-1 compared to samples with low expression of HMOX-1 in a TCGA melanoma cohort (406 samples with survival information) (**A**) and in *BRAF* mutant samples (165 samples) (**B**) HMOX-1 expression was stratified as “high” and “low” with an optimized cut-off. (**C**) Boxplot representing the distribution of HMOX-1 expression in sensitive and resistant mice after A375 cell inoculation and BRAF inhibitor treatment (GSE74729, 4 sensitive and 5 resistant samples). (**D**) Boxplot representing the distribution of HMOX-1 expression in sensitive and resistant patients in a merge of 3 melanoma patient datasets (43 BRAF inhibitor sensitive (before treatment) and 48 resistant and relapse of disease (after treatment)). FC, fold change; DFS, disease-free survival.

Moreover, *HMOX-1* overexpression was associated with lower disease-free survival (*p* = 0.0004), as depicted in (Figure 2A). Samples with a high expression of *HMOX-1* were 19% more likely to present a disease relapse at 5 years compared to samples with lower expression. A Cox regression model validated the previous survival analysis, where high HMOX-1 was associated with a higher rate of recurrence (hazard ratio (HR) = 1.55, 95% CI = 1.21–1.99, *p* = 5.55 × 10^−4^).

Interestingly, HMOX-1 expression also significantly stratified the patient outcome in *BRAF^V600E^* mutant melanoma samples (log-rank *p* = 0.03), as shown in Figure 2B (HR = 1.54, 95%CI = 1.05–2.27, *p* = 0.027).

Finally, public transcriptomic databases were used to validate the role of the HMOX-1 gene in response to BRAF inhibitor treatment in melanoma xenograft models and patients.

HMOX-1 expression was collected from a cohort of A375 xenograft mice treated with the BRAF inhibitor. As shown in Figure 2C, HMOX-1 was higher in resistant samples than in sensitive samples (*p* = 0.005). Furthermore, in an in vitro model of an A375 cell line treated with a BRAF inhibitor, HMOX1 was also found to be upregulated in the resistant clones, harboring a spontaneous NRAS mutation, compared to the sensitive clones (*p* = 0.08) (Figure S9B). This observation was confirmed in a patient cohort treated with BRAF inhibitors. HMOX-1 expression was higher in resistant samples than in sensitive samples (*p* = 0.012) (Figure 2D).

### 3.3. Translocation of BRAF^V600E^ to the Nucleus Promotes HMOX-1 Upregulation in a Xenograft Mouse Model of Melanoma

**Figure 3:**
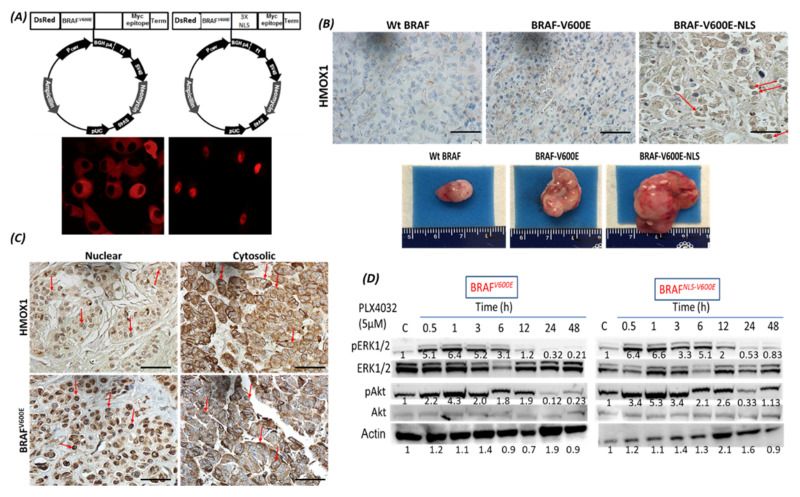
Nuclear BRAF^V600E^ and HMOX-1 expression in xenograft mouse and human melanoma tissue cores. (**A**) The plasmid (*BRAF^V600E^*) or (3XNLS-*BRAF^V600E^*), containing BRAF^V600E^ with nuclear localization sequences (NLS), was transfected into the melanoma cell line MV3 (*BRAF^wt/wt^*) and selected with antibiotic G418. (**B**) MV3 cells transduced with BRAF^V600E^, NLS-BRAF^V600E^, and control cells were injected in mice (*n* = 5 for each group), and representative tumor images were taken. Xenograft mouse tissues were stained with an anti-HOMX1 antibody. Mag. 400×. Scale bars = 50 μm (**C**) Human malignant melanoma tissue array: immunoreactivity to BRAF^V600E^ and HMOX1 in the human melanoma cores was detected and counterstained for the nuclear and cytoplasm location. Mag. 400×. Scale bars = 50 μm. (Figure S3) provides the whole IHC analysis. (**D**) phosphoERK and phosphoAKT were assessed in MEF stably transfected with BRAF^V600E^ or NLS-BRAF^V600E^ after treatment with 5 μM PLX-4032 at different time points. A representative gel is shown. Two additional independent experiments provided similar results.

First, we constructed a lentiviral vector in which *BRAF^V600E^* was inserted in-frame with a sequence encoding 3 NLS domains followed by DsRed2, which encodes a fluorescent protein. The mutant kinase, NLS-BRAF^V600E^/DsRed2, was localized exclusively in the nucleus (Figure 3A, right). *BRAF^V600E^* was also cloned into the DsRed2 vector without NLS, to be localized exclusively in the cytoplasm, as shown in Figure 3A (left). A vector expressing NLS/DsRed2 was also generated to serve as a control. Melanoma MV3 cells, which express *WT BRAF*, were transduced with the three different viral vectors. After antibiotic selection, the expression of BRAF^V600E^ was confirmed by immunofluorescence analysis (Figure 3A).

Next, we used a mouse xenograft model of melanoma to examine whether overexpression of nuclear BRAF^V600E^ upregulates HMOX-1 protein expression. Stable clones of melanoma MV3 cells expressing BRAF^V600E^ or NLS-BRAF^V600E^ were subcutaneously inoculated into nude female mice (*n* = 5 for each group), and the animals were monitored for 5 weeks. Figure 3B and Figure S10 show that tumors generated by the BRAF^V600E^- and NLS-BRAF^V600E^-expressing MV3 cells were greater in size and displayed higher HMOX-1 expression compared to the wild-type control, as assessed by immunohistochemistry with a specific antibody to the mutant protein. As expected, Figure 3B shows that the NLS-expressing cells generated the largest size of tumor and much higher HMOX-1 protein. In BRAF^V600E^-injected mice (*n* = 5), HMOX-1 was only detected in 2 out of 5 (40%), while in NLS-BRAF^V600E^-injected mice (*n* = 5), HMOX-1 was detected in 4 out of 5 mice (80%). This result suggests that HMOX-1 protein expression could be mediated by the nuclear BRAF^V600E^.

### 3.4. The Localization of BRAF^V600E^ and HMOX-1 in Human Melanoma Samples

To test our hypothesis that nuclear BRAF^V600E^ enhances HMOX-1 protein expression, we analyzed 30 tissue cores of human cutaneous and metastatic melanomas (15 cores each) for protein expressions of BRAF^V600E^ and HMOX-1 using the IHC technique (Figure 3C). The expression of nuclear BRAF^V600E^ was 33.3% (5 out of 15 cores) in both cases. Interestingly, 40% of metastatic cores had HMOX-1 expression in the nucleus (6 out of 15 cores) compared to 20% of cutaneous cores (3 out of 15 cores). All metastatic cores with nuclear BRAF^V600E^ staining were positive for nuclear HMOX-1, while only 60% of primary cores with nuclear BRAF^V600E^ localization had nuclear HMOX-1 staining. The results of the whole cohort are summarized in the Figure S3. We next examined whether nuclear localization of BRAF^V600E^ affected the pattern of ERK and Akt phosphorylation in mouse embryonic fibroblasts (MEFs). We used these MEFs because they are *BRAF* knockout (Figure S6). Figure 3D shows that NLS-BRAF^V600E^-harboring MEFs compared to control cells displayed higher levels of ERK phosphorylation after 6 h and a persistent pERK up to 12 h of treatment with PLX-4032. Treatment with PLX-4032 of BRAF^V600E^-harboring MEF cells induced transient feeble phosphorylation of ERK at 12 h. After 0.5 to 12 h, the phosphorylation pattern of Akt in NLS-BRAF^V600E^-expressing cells remained much higher than those observed in similarly treated BRAF^V600E^-expressing cells. Interestingly, at 48 h of PLX-4032 treatment, the AKT pathway is reactivated in NLS-BRAF^V600E^-expressing cells. This is a confirmation of the phosphoERK and phosphoAKT patterns found in our previous study using MV3 cells [24].

### 3.5. Suppression of HMOX-1 Has an Anti-Proliferative Effect in Resistant Melanoma Cells

**Figure 4:**
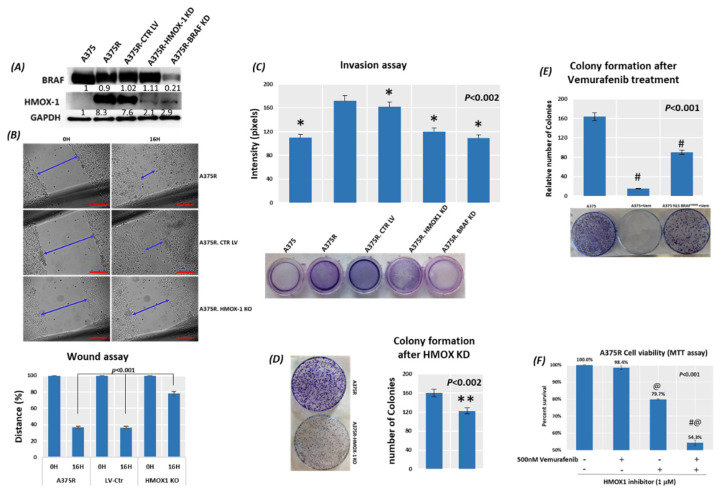
Suppression of HMOX-1 has an anti-proliferative effect in a resistant A375 (R) cell line. (**A**) The protein levels of BRAF^V600E^ and HMOX-1 were measured in A375P and A375R cells with a knockdown of *BRAF^V600E^* or HMOX-1. Cell migration or invasion was evaluated by using a wound-healing assay (**B**) or invasion assay Scale bars = 200 μm. 40× magnification. All assays were performed in triplicate. * *p* < 0.002 compared with A375R (**C**) in A375R cells with knockdown of HMOX-1. (**D**) Representative colony formation of A375R cells with HMOX-1 knockdown. The assay was done in triplicate. ** *p* < 0.002 compared with A375R. (**E**) Colony formation of drug-sensitive A375 cells expressing NLS- BRAF^V600E^ treated with 500 nM vemurafenib. # *p* < 0.001 compared with A375. (**F**) A375R cell viability was evaluated by MTT assay with the HMOX-1 inhibitor (OB-24) and/or in combination with vemurafenib. Means ± SEM are shown. @ *p* < 0.001 compared with control and # *p* < 0.001 compared with the treated HMOX1 inhibitor.

We first established stable A375R cell lines with HMOX-1 knockdown by using HMOX-1 shRNA lentiviral particles, as recommended by Santa Cruz’s protocol. Next, we selected stable clones that express shRNA via puromycin dihydrochloride. To verify HMOX-1 knockdown and its effect on cell viability, we performed Western blot analyses and an apoptosis assay (Figure 4A and Figure S2). The HMOX-1 knockdown, compared to the parental A375R cells, did not induce apoptosis before the treatment with vemurafenib (Figure S2). In line with a previous study [27], *BRAF^V600E^* knockdown reduced HMOX-1 protein expression in A375R cells (Figure 4A). In a wound-healing assay, we found that the silencing of HMOX-1 reduced cell proliferation by about 3-fold (Figure 4B). Since the number of cells in the denuded area rises either due to immigration of cells from the wound edge or by mitosis of the migrated cells, the contribution of cell migration to the increase of the cell population in the denuded space was determined after inhibiting cell division with an antimitotic agent, Vindoline (Figure S1). No difference was observed before or after Vindoline treatment. Within the 16 h of the wound healing assay, the denuded area was mostly covered up by the migrating cells. Moreover, invasion ability was dramatically reduced in the HMOX-1 knockdown cells and practically eradicated in the *BRAF* knockdown cells (Figure 4C). The colony formation assay with A375R showed a reduced ability of a single cell to grow into a colony when HMOX-1 was knocked down (Figure 4D). Additionally, drug-sensitive A375 cells expressing NLS-BRAF^V600E^ were more resistant to vemurafenib treatment and were able to grow into colonies compared to control parental cells (Figure 4E). Interestingly, the MTT assay revealed that cell viability in A375R cells was clearly diminished after treatment with the specific HMOX-1 pharmacologic inhibitor OB-24 in combination with vemurafenib, suggesting that HMOX-1 is the predominant mediator of cell aggressiveness in the presence of nuclear BRAF^V600E^ (Figure 4F). These data show that HMOX-1 can promote melanoma cell proliferation. The validation of this finding with a different melanoma cell line was performed with WM983B cells (Figure S5).

### 3.6. Suppression of HMOX-1 Reduces the Number and Size of Tumors in a Preclinical Melanoma Model

Furthermore, we explored the role of HMOX-1 in promoting melanoma aggressiveness using a xenograft mouse model. The results show that the number, size, and weight of tumors significantly decreased in mice receiving HMOX-1-knockdown cells.

**Figure 5:**
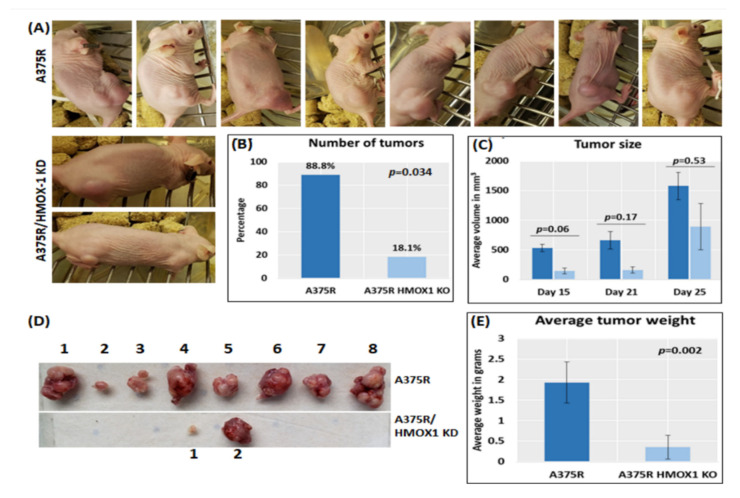
HMOX-1 knockdown reduces tumor growth in a melanoma mouse model. Mice were subcutaneously injected with A375R or A375R with HMOX-1 knockdown (2 × 10^6^ cells/mouse). (**A**) Mice with visible tumors of each group. (**B**) Number of tumors per group. (**C**) Tumor size was monitored twice a week for 4 weeks. (**D**) Representative tumor images were taken from the HMOX-1 knockdown group and the control group. (**E**) The tumor weight was recorded at the end of the experiment (day 25).

A375R cells with HMOX-1 knockdown or the parental cells were subcutaneously inoculated into nude female mice. Two groups of mice were used: *n* = 9 for the A375R cells and n = 11 for the HMOX-1 knockdown cells. Tumors started to develop after 19 days and were monitored twice a week for an extra 14 days. The results show that almost 90% of the A375R-injected mice developed a tumor (8 out of 9) compared to about 20% of the HMOX-1 knockdown mice (2 out of 11), as shown in Figure 5A,B. The growth rate of the tumors generated by HMOX-1 knockdown A375R cells was markedly slower than those expressing the A375R parental cells (Figure 5C). In addition, the average weight of tumors dropped in HMOX-1-knockdown-injected mice from 1.92 to 0.25 g (Figure 5D,E).

### 3.7. Pathways Analysis Revealed the Functional Role of HMOX1 in the BRAF Inhibitor Resistance Process

**Figure 6:**
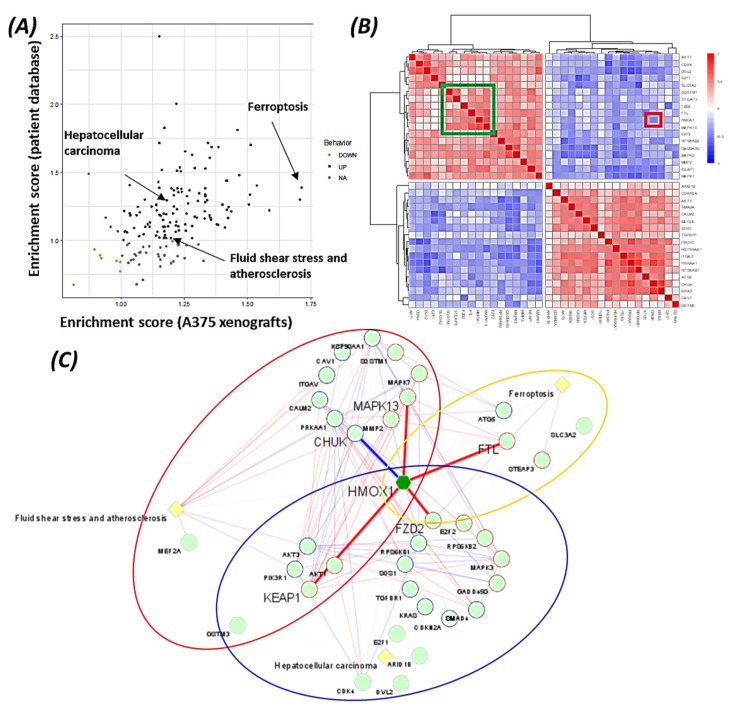
Pathway analysis revealed a key role of HMOX-1 in BRAF inhibitor therapy resistance in the xenograft model and in melanoma patients. (**A**) Dotplot representing the enrichment score obtained for the common pathways identified in the xenograft model and melanoma patients. (**B**) Heatmap representing the correlation matrix between the 36 genes involved in the 3 selected pathways (ferroptosis, fluid shear stress and atherosclerosis, and hepatocellular carcinoma) common in the xenograft model and melanoma patients. Values are from the patient dataset. (**C**) Network representation of the 36 genes (green nodes) involved in the 3 selected pathways (light yellow nodes), with their Spearman correlation as edges. Dark red ellipse, genes belonging to “fluid shear stress and atherosclerosis”; orange, genes belonging to “ferroptosis”; blue ellipse, genes belonging to “hepatocellular carcinoma”; thicker edges, correlation related to HMOX1; blue edges, negative correlation; red edges, positive correlation; edge transparency proportional to correlation value; blue node border, negative correlation with HMOX1; red node border, positive correlation with HMOX1. Correlation values are from the patient dataset.

KEGG pathways analysis was independently performed on the A375 xenograft database and the melanoma patient database, and 161 signaling pathways were common in both databases (Figure 6A). Interestingly, three pathways contained *HMOX-1*, “ferroptosis”, “fluid shear stress and atherosclerosis”, and “hepatocellular carcinoma.” Ferroptosis is a newly discovered iron-dependent cell death process. Fluid shear stress and atherosclerosis are diseases characterized by the deregulation of PI3-AKT signaling, focal adhesion, NF-kappa B signaling, and MAPK signaling pathways. Finally, hepatocellular carcinoma is a cancer involving calcium, PI3K/AKT, p53, TGF-beta, Wnt, and MAPK signaling pathways. Interestingly, ferroptosis was the pathway with the highest enrichment score in xenografts and the only one to show a significant correlation with *HMOX-1* in both databases (xenografts, rho = 0.79, *p* = 0.02; patients, rho = 0.39, *p* = 0.002). These observations suggest that HMOX-1 contributed significantly to the ferroptosis process during BRAF inhibition therapy resistance. From the three pathways containing *HMOX-1*, 36 genes were identified as significantly deregulated in the resistant models, compared to sensitive samples, in both databases. The 36 genes were enriched in the PI3K-AKT pathway (*AKT1*, *AKT3*, *PI3KR1*, *PRKAA1*, *RPS6KB1*, *RPS6KB2*), the MAPK pathway (*KRAS*, *SOS1*, *MAPK13*, *MAPK3*, *MAPK7*, *MEF2A*), iron metabolism (*HMOX1*, *FTL*, *ATG5*, *STEAP3*), the chromatin remodeling complex (*ARID1B)*, cell cycle (*CDK4, CDKN2A, E2F1, E2F2)*, the NF-kappa B pathway (*CHUK, ITGAV)*, the WNT pathway (*DVL2, FZD2*), TGF-beta pathway (*SMAD4, TGFBR1*), and the antioxidative response (*HMOX-1, GSMT3, KEAP1, SQSTM1*). A correlation matrix (Figure 6B) identified a cluster of genes strongly positively associated with *HMOX-1* (FTL and STEAP3, two ferroptosis genes, FZD2, MAPK13, and SQSTM1; highlighted with the green square on the heatmap) and one gene strongly negatively correlated (CHUK, with a red square on the heatmap). A network analysis emphasized the interdependence of the three pathways involving *HMOX-1* (Figure 6C). The highest correlations observed with *HMOX-1* in patients with melanoma were with FZD2 (rho = 0.54), KEAP1 (rho = 0.59), MAPK13 (rho = 0.68), the “ferroptosis” gene FTL (rho = 0.58), and CHUK (rho = −0.50).

The data suggest that *HMOX-1* actively participates in BRAF inhibitors’ resistance process, linking different signaling pathways that are known to be involved in therapy resistance (PI3K, MAPK, TGF-beta, and Wnt pathways).

## 4. Discussion

BRAF is a proto-oncogene (also known as v-raf murine sarcoma viral homolog B1) that belongs to the Raf kinase family [28]. *BRAF^V600E^* mutations are linked with several types of tumors, where they activate the MAPK signaling pathway constitutively, resulting in uncontrolled cell proliferation and survival [29].

Transport across cell compartments is required for functional regulation and for diversity in the cell, but aberrant nucleocytoplasmic trafficking has been implicated in cancers such as thyroid, melanoma, and others [30,31,32,33].

**Figure 7:**
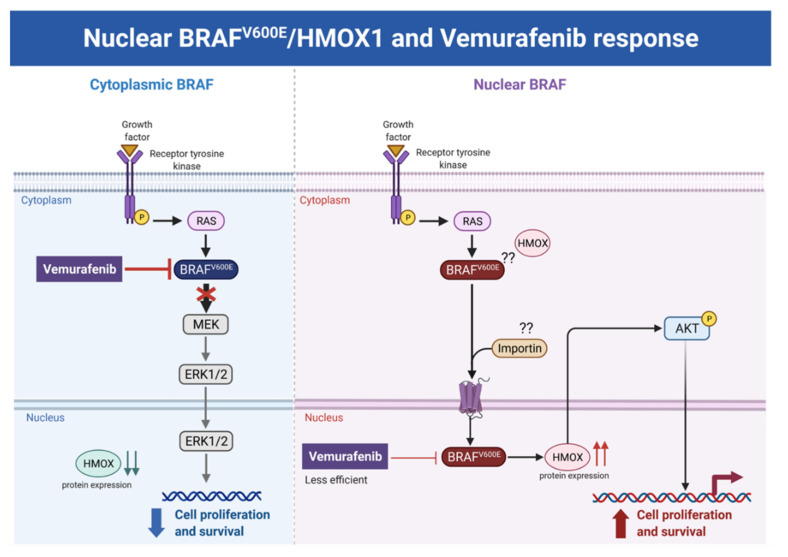
Hypothetical model of the melanoma resistance to vemurafenib. Melanoma cells with cytoplasmic BRAFV600E and low HMOX-1 expression are more sensitive to vemurafenib treatment than cells with nuclear BRAFV600E, high HMOX-1 expression, and an activated AKT pathway.

We reported for the first time in our previous study [24] a strong association between nuclear BRAF^V600E^ expression and aggressive clinicopathologic features, including overall stage, tumor stage, lymph node metastasis, depth of invasion, Clark level, mitotic activity, and ulceration. In the same study, we reported that the SkMel-28 cell line (*BRAF^V600E^*) displays a dynamic nuclear localization of BRAF^V600E^, regulated by the removal or addition of FBS or epidermal growth factor (EGF) to the media. In contrast, and as we report in the current study (Figure S11), WT BRAF does not show any nuclear localization by immunofluorescence in the SKMel-202 melanoma cell line. In the literature, two studies mentioned a small fraction of WT BRAF in the nucleus. In the first study, an exogenous truncated BRAF transfected in NIH3T3 cells was used, and this artificial system cannot be generalized or applied to the endogenous BRAF. In the second study, Shin et al. used a non-cancer C2C12 myoblast cell line and found a very tiny fraction of WT BRAF in the nucleus compared to a strong nuclear presence of BRAF^V600E^ [34,35]. Our hypothesis is that WT BRAF activation requires external growth factors, and its localization is totally cytoplasmic. The constitutively active BRAF^V600E^ is independent of external stimulus, and the nuclear localization may provide the BRAF^V600E^ with a sheltering mechanism against inhibitors, possibly through a novel and unexpected collaboration with overexpressed HMOX1. However, mechanistic studies are required for the validation and explanation of our previous findings. In this study, we report that A375R melanoma vemurafenib-resistant cells display a nuclear localization of BRAF^V600E^ after synchronization and stimulation with the FBS. A 10-fold upregulation of HMOX-1 protein is observed in A375R cells with nuclear localization of BRAF^V600E^ compared to A375 parental cells that have cytoplasmic BRAF^V600E^. Surprisingly, HMOX1 upregulation in A375R or WM983C cell lines is not the consequence of NRF2 protein overexpression (Figure S7). In another study, it was hypothesized that the existence of a possible HMOX-1 reserve in the form of mRNA to be transformed into protein when necessary [36]. Additionally, we show that HMOX-1 upregulation crucially limits the efficacy of the BRAF inhibitor PLX4032 and is important in promoting cell viability and aggressiveness. Our results provide the first evidence that nuclear BRAF^V600E^ plays a critical role in the regulation of HMOX-1 protein overexpression in resistant melanoma cells. A recent study with a *WT BRAF* cell line (MeWO) showed neither basal expression nor induction of HMOX-1 after treatment with PLX4032 [37]. HMOX-1 is one of the most important mechanisms of cell adaptation to stress, and chemo- and radiotherapy both fundamentally stimulate HMOX-1 expression [38]. However, the transcriptional or post-transcriptional mechanisms controlling HMOX-1 expression in response to cytotoxic stress remain elusive. This study extends our understanding by identifying nuclear BRAF^V600E^ as a potential player in HMOX-1 expression. The nuclear translocation and the molecular mechanism of nuclear BRAF^V600E^ in promoting tumor progression and resistance is still poorly understood. Thus, to the best of our knowledge, the present study provides the first evidence of a possible role of nuclear BRAF^V600E^/HMOX-1/AKT in melanoma resistance. Figure 7 is a hypothetical model of how melanoma cells with nuclear BRAF and high HMOX-1 expression can activate the AKT pathway and resist vemurafenib treatment. 

Using bioinformatics analysis, we determined a relationship between HMOX-1 upregulation and disease-free survival (DFS). Indeed, patients with high HMOX-1 have a 19% lower chance of survival after five years than patients with lower HMOX-1 expression. The resistance to BRAF^V600E^ inhibitors is also correlated with the high expression of HMOX-1 in a melanoma mouse model and in a melanoma patient database. 

To further corroborate our bioinformatics results, cells expressing nuclear and cytoplasmic BRAF^V600E^ were subcutaneously injected into nude mice. Nuclear BRAF^V600E^ cells were able to induce more HMOX-1 protein expression and phosphorylated Akt than cells harboring cytoplasmic BRAF^V600E^. Following the same pattern, the human metastatic melanoma cores expressed more nuclear HMOX-1 when BRAF^V600E^ was detected in the nucleus. This nuclear HMOX-1 expression in nuclear BRAF^V600E^ cores was more prevalent in metastatic malignant melanoma specimens. Remarkably, the intranuclear localization of the kinase raised the resistance of melanoma cells to drug therapy, and this was supported by reactivation of the Akt pathway as an alternative pathway for cell survival. Thus, targeting mediators of Akt activation could be a possible option for intervening with drug resistance in metastatic melanoma [39].

To address whether HMOX-1 reduction could interfere with melanoma aggressiveness, compromised HMOX-1 cell lines have been established and are correlated with melanoma-decelerated cell proliferation and invasion rate along with slower colony formation. Furthermore, an in vivo study showed a significant decrease in the number, size, and weight of tumors after knocking down HMOX-1. Remarkably, the A375 sensitive cells developed resistance when expressing NLS-BRAF^V600E^.

Interestingly, the pathway analysis showed that ferroptosis is highly associated with HMOX-1 expression. Ferroptosis is an iron-dependent cell death process characterized by the accumulation of lipid peroxides and is genetically and biochemically different from apoptosis. It is worth noting that nuclear factor (erythroid-derived 2)-like 2 (NRF2) activation and the subsequent deregulation of iron signaling in cancers have been implicated in cancer development. Constitutive NRF2 activation and NRF2-dependent upregulation of the iron storage protein ferritin (FTL) or HMOX-1 can lead to enhanced proliferation and therapy resistance [40,41,42].

## 5. Conclusions

In conclusion, there is increasing interest in a possible means of inhibiting HMOX-1 expression in order to improve the sensitivity of cancer cells to BRAF^V600E^ inhibitors. In this context, the nuclear BRAF^V600E^/HMOX-1/AKT axis is associated with melanoma aggressiveness. The cytoplasmic BRAF^V600E^ expression had a modest effect in promoting HMOX-1 overexpression and was not correlated with advanced disease. Therefore, the combination of specific HMOX-1 inhibitors with BRAF^V600E^ inhibitors is anticipated to reduce melanoma aggressiveness and improve BRAF^V600E^ inhibitor-based therapies.

## Data Availability

The datasets generated and/or analyzed in this study are available upon reasonable request.

## Supplementary Materials

The following supporting information can be downloaded at: https://www.mdpi.com/article/10.3390/cancers14020311/s1, Figure S1: Cell migration; Figure S2: Apoptosis; Figure S3: Human melanoma cores; Figure S4: List of 84 cancer related proteins; Figure S5: Suppression of HMOX1 in WM983B; Figure S6: BRAF V600E and BRAF V600E -NLS expression in MEF -/- BRAF cells; Figure S7: NRF2 expression; Figure S8: Cell fractionations from SKMel-28 cells; Figure S9: mRNA HMOX1 expression; Figure S10: Tumors size; Figure S11: BRAF localization in SKMel-28 and SKMel-202 cells.
