# Supplementary material for: Nuclear Localization of BRAFV600E Is Associated with HMOX-1 Upregulation and Aggressive Behavior of Melanoma Cells
Source: Cancers (Basel). 2022 Jan 9;14(2):311. doi: 10.3390/cancers14020311 (PMC8773521; doi:10.3390/cancers14020311)
Supplement: Supplementary file 1 [file cancers-14-00311-s001.zip › cancers-1450741-supplementary/cancers-1450741-supplementary Figures.pdf]

Supplementary materials

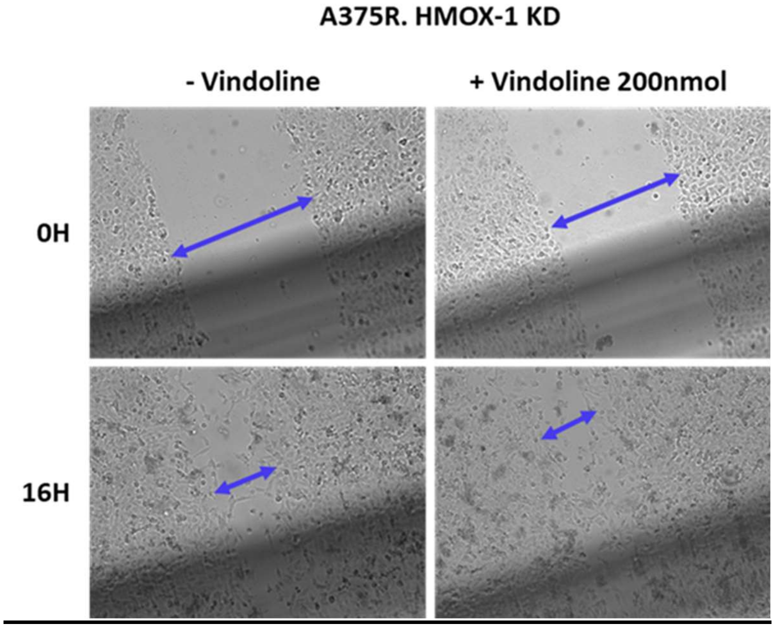

Figure S1. Cell migration was evaluated by using wound healing assay in presence or absence of an anti-mitotic agent. No difference was observed before and after Vindoline treatment. No contribution of cell division to the increase of the cell population in the denuded space.

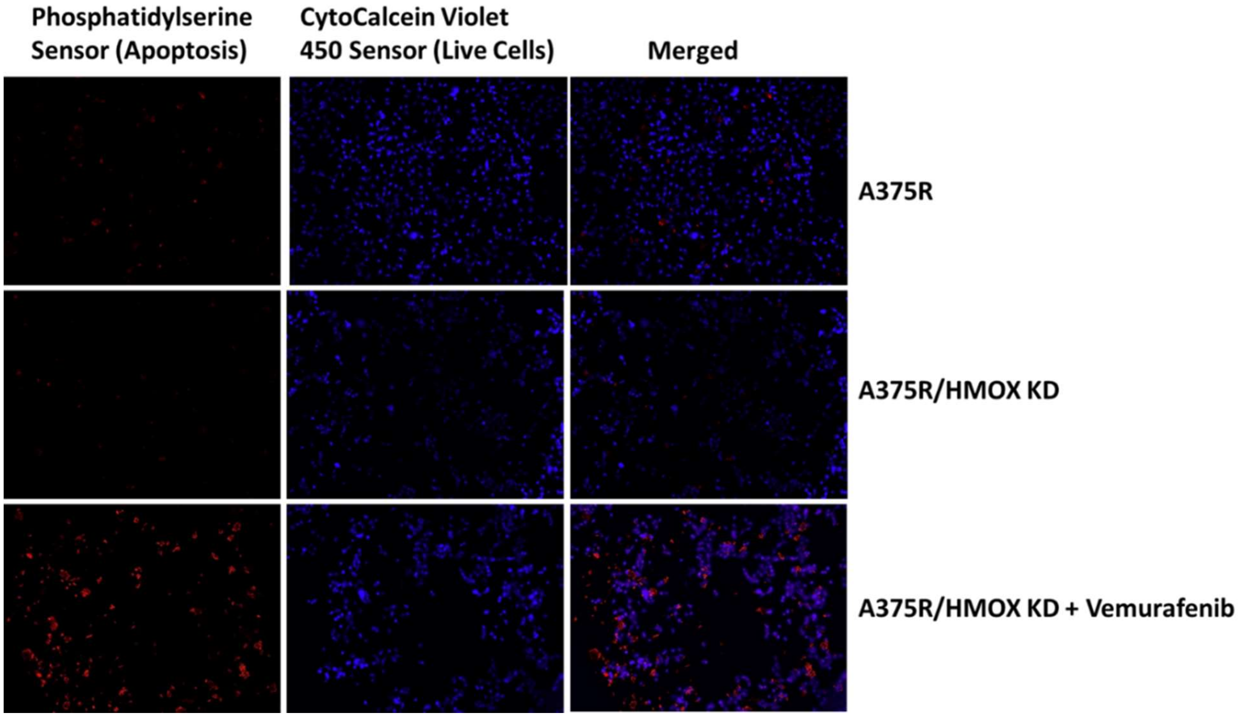

Figure S2. Measurement of apoptosis in A375R and A375R/HMOX-1 KD cell exposed to vemurafenib treatment. HMOX knock down (KD) does not initiate apoptosis before vemurafenib treatment.

		HMOX-1	BRAFV600E
Primary malignant melanoma	#Cores	15	15
	Cytosolic (%)	13 (86.7)	14 (93.3)
	Nuclear (%)	3 (20)	5 (33.3)
Metastatic malignant melanoma	#Cores	15	15
	Cytosolic (%)	13 (86.7)	15 (100)
	Nuclear (%)	6 (40)	5 (33.3)

Figure S3. Localization of $BRAF^{V600E}$ and HMOX-1 in human melanoma cores. Table summarizing the number of samples with cytoplasmic or nuclear $BRAF^{V600E}$/HMOX-1 after IHC analysis.

AFP	ErbB4	MMP-2
Amphiregulin	FGF basic	MMP-3
Angiopoietin-1	FoxC2	MMP-9
ANGPTL4	FKHR	MSP/MST1
ENPP-2/Autotaxin	Galectin-3	MUC-1
AXL	GM-CSF	Nectin-4
BCL-X	HCG	Osteopontin
CA125/MUC-16	HGF R/c-Met	p27/Kip1
E-Cadherin	HIF-1alpha	p53
VE-Cadherin	HNF-3beta	PDGF-AA
CAP-G	HO-1/HMOX1	CD31/PECAM-1
CA-9	ICAM-1/CD54	Progesterone R
Cathepsin B	CD25/IL-2 R alpha	Progranulin
Cathepsin D	IL-6	Prolactin
Cathepsin S	CXCL8/IL-8	Prostasin
CEACAM-5	IL-18 Bpa	E-Selectin
Decorin	KLK-3/PSA	Maspin
DKK-1	KLK-5	PAI-1/Serpin E1
DLL-1	KLK-6	SNAIL
EGF R/ErbB1	Leptin (OB)	SPARC
Endoglin/CD105	Lumican	Survivin
Endostatin	CCL2/MCP-1	Tenascin-C
Enolase 2	CCL8/MCP-2	THBS-1
eNOS	CCL7/MCP-3	TIE-2
EpCAM	M-CSF	UPA-1
ER-alpha	Mesothelin	VCAM-1
ErbB2	CCL3/MIP-1alpha	VEGF
ErbB3	CCL20/MIP-3alpha	Vimentin

Figure S4. The full list of the 84 cancer related proteins array.

https://www.rndsystems.com/products/proteome-profiler-human-xl-oncology-array_ary026

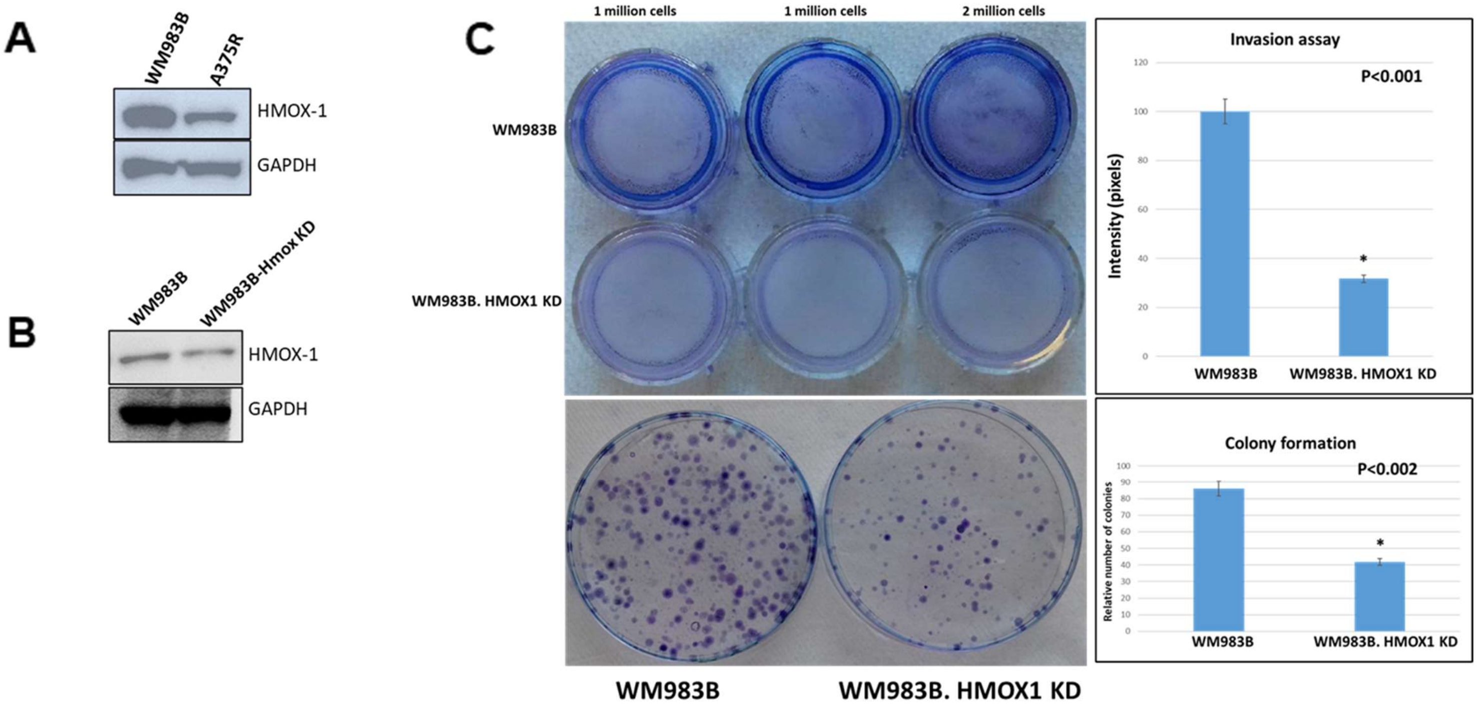

Figure S5. Suppression of HMOX-1 has an Anti-Proliferative Effect in WM983B Melanoma Cells. **(A)** HMOX1 expression in two resistant cell lines, A375R and WM983B. **(B)** HMOX1 knock down in WM983B cell line. **(C)** Invasion and colony formation assays before and after HMOX1 knock down in WM983B cells

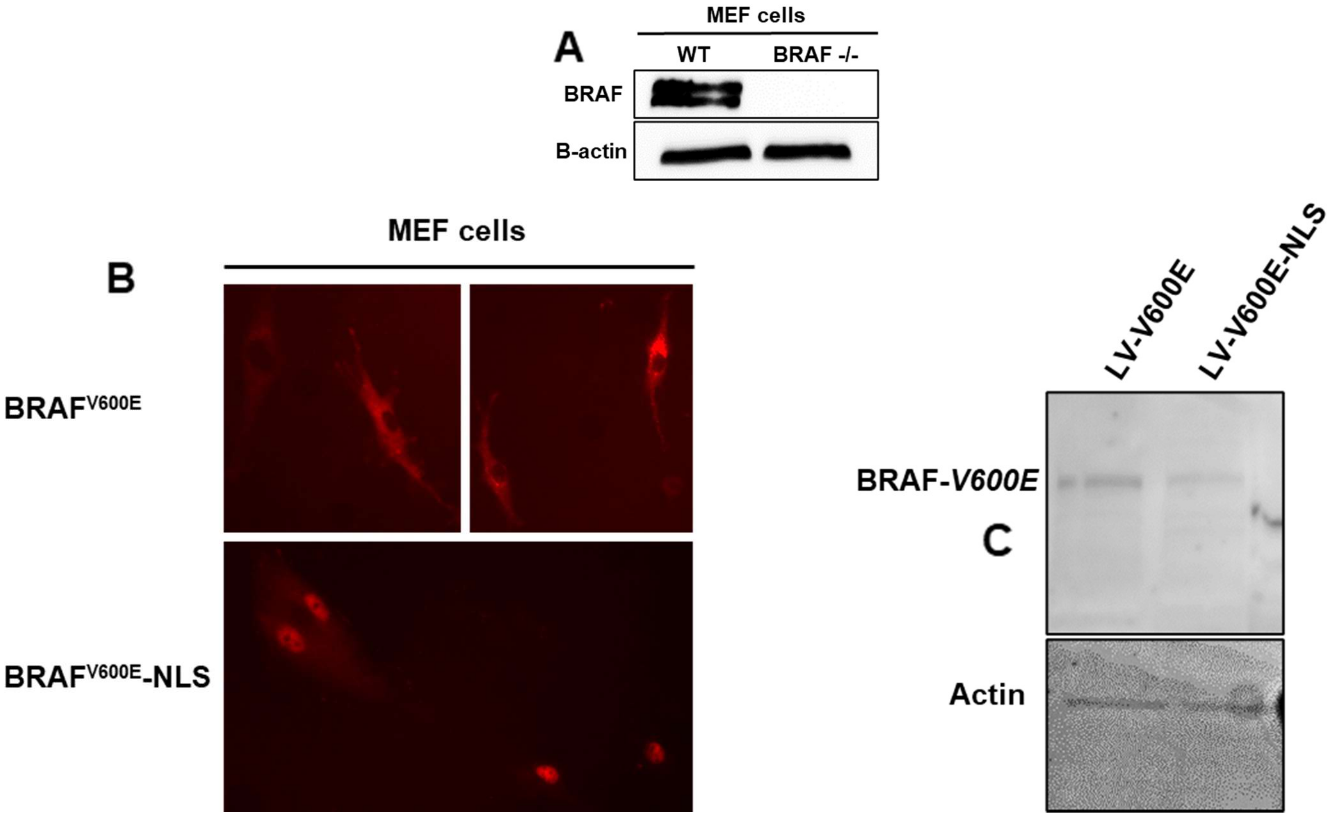

Figure S6. BRAF V600E and BRAF V600E -NLS expression in MEF -/- BRAF cells. **(A)** BRAF expression in WT and BRAF-/- MEF. **(B)** LV-BRAF^{V600E} and LV-BRAF^{V600E}-NLS expression in MEF BRAF-/- assessed by Immunofluorescence **(C)** LV-BRAF^{V600E} and LV-BRAF^{V600E}-NLS expression in MEF BRAF-/- assessed by WB.

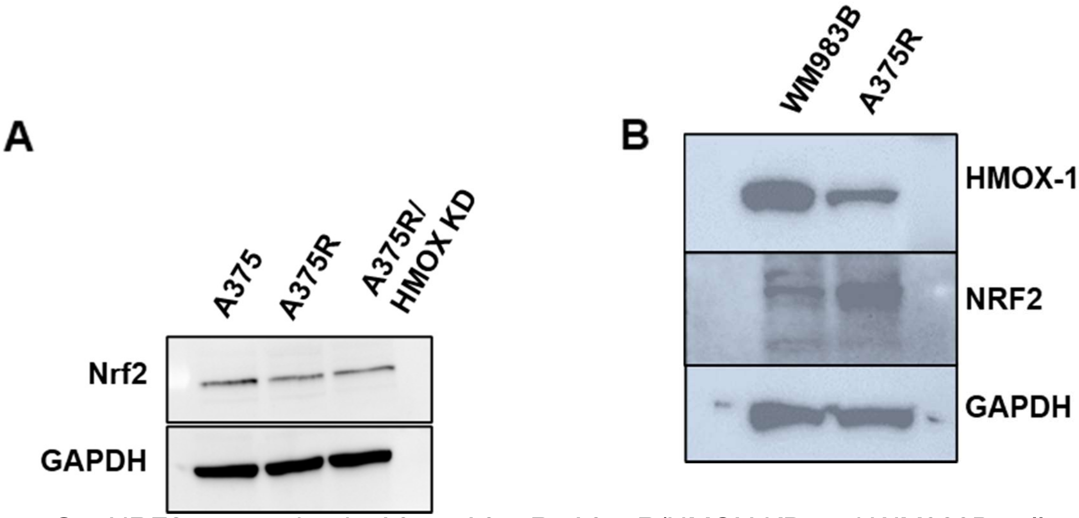

Figure S7. NRF2 expression in A375, A375R, A375R/HMOX KD and WM983B cells. **(A)** NRF2 expression in A375, A375R and A375R/HMOX1KD. **(B)** NRF2 expression in A375R and WM983B

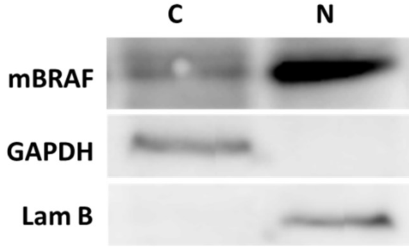

Figure S8. Cell fractionations from SkMel-28 cells (**C**: cytosolic; N: nuclear) were prepared and subjected to western blot analysis using anti-BRAFV600E (VE1 clone), Anti-GAPDH and anti-Lamin B antibodies

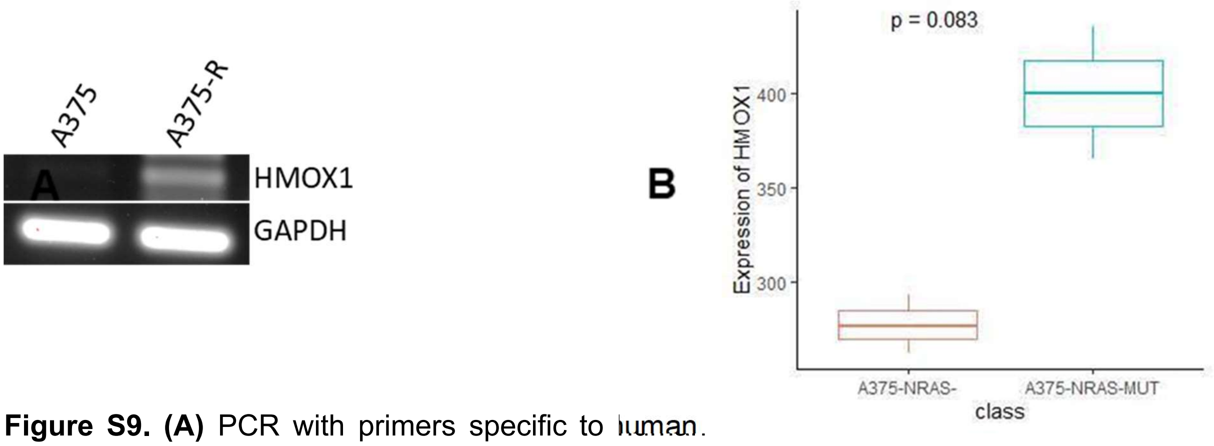

Figure S9. (A) PCR with primers specific to human.
HMOX-1 or GAPDH. **(B)** *In vitro* model of A375 cell line treated with a BRAF inhibitor, HMOX1

was upregulated in the resistant clones, harboring a spontaneous NRAS mutation, compared to the sensitive clones (p=0.08). GSE74729 GEO dataset (https://www.ncbi.nlm.nih.gov/gds).

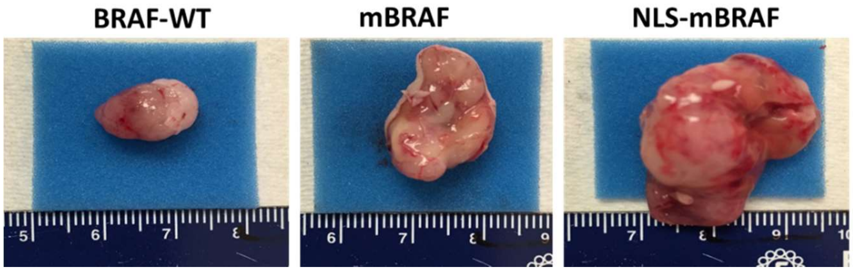

Figure S10. Tumors from mice subcutaneously injected with MV3 cells with either nuclear localization signal (NLS)-BRAFV600E or BRAFV600E.

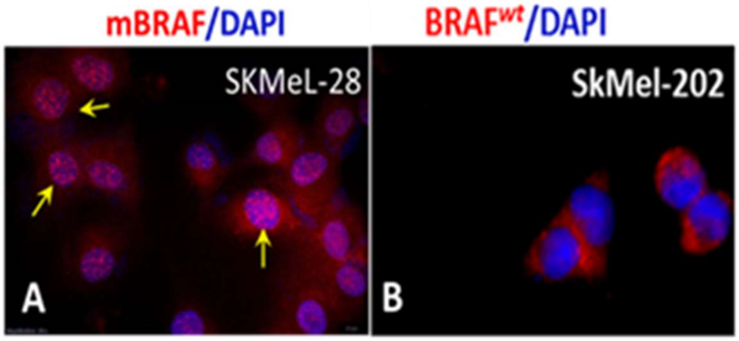

Figure S11. Nuclear localization of WT BRAF and BRAF V600E in melanoma cells. SkMel-28 and SkMel-202 Cells were grown in a complete RPMI medium, fixed with 4% formalin and stained with VE1 (SkMel-28) or Pan-BRAF (SkMel-202) antibodies and visualized by florescence Microscopy. Arrows indicate the nuclear localization of BRAF V600E; Mag: 400X.
