# Supplementary material for: Nuclear Localization of BRAFV600E Is Associated with HMOX-1 Upregulation and Aggressive Behavior of Melanoma Cells
Source: Cancers (Basel). 2022 Jan 9;14(2):311. doi: 10.3390/cancers14020311 (PMC8773521; doi:10.3390/cancers14020311)

Original blots for Fig. 3D

MEF Cells

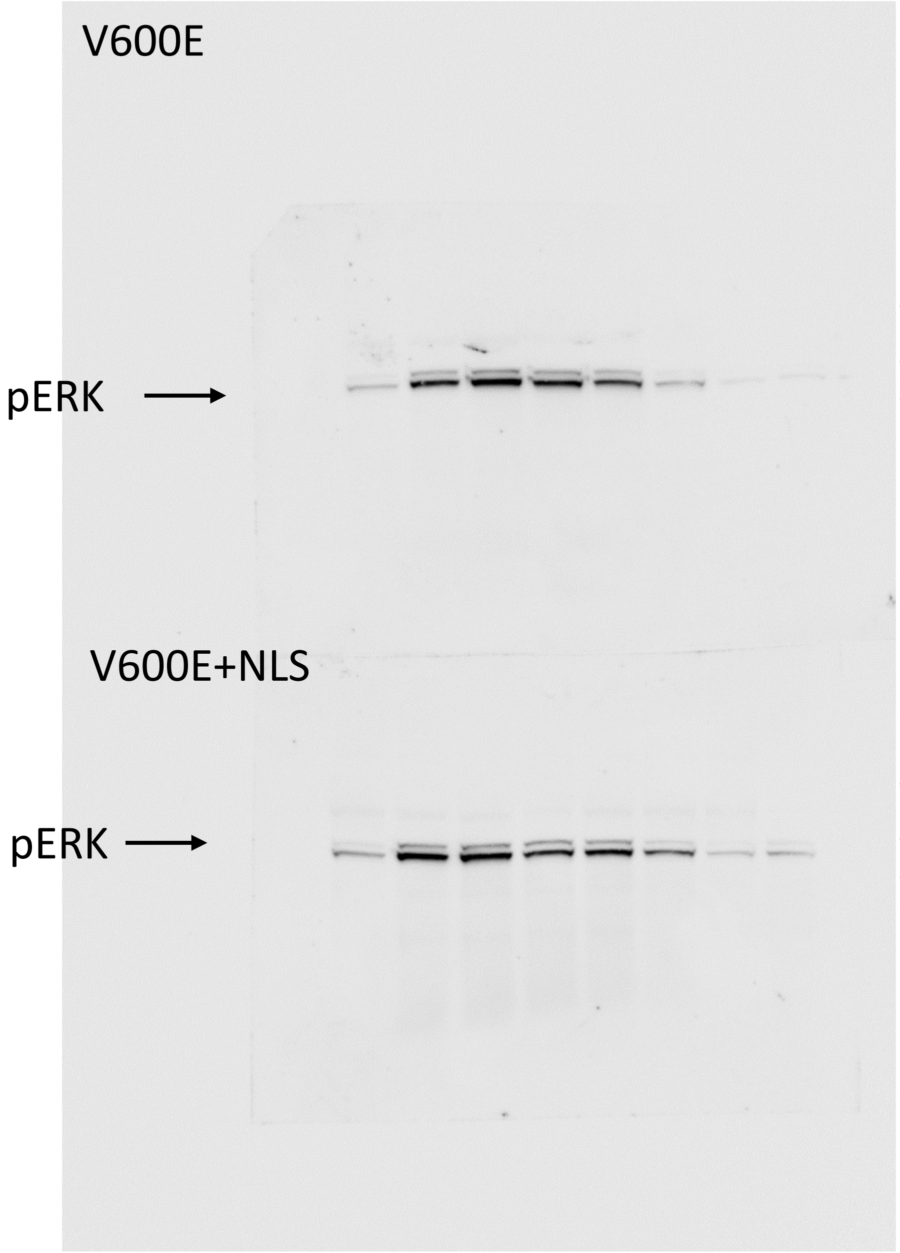

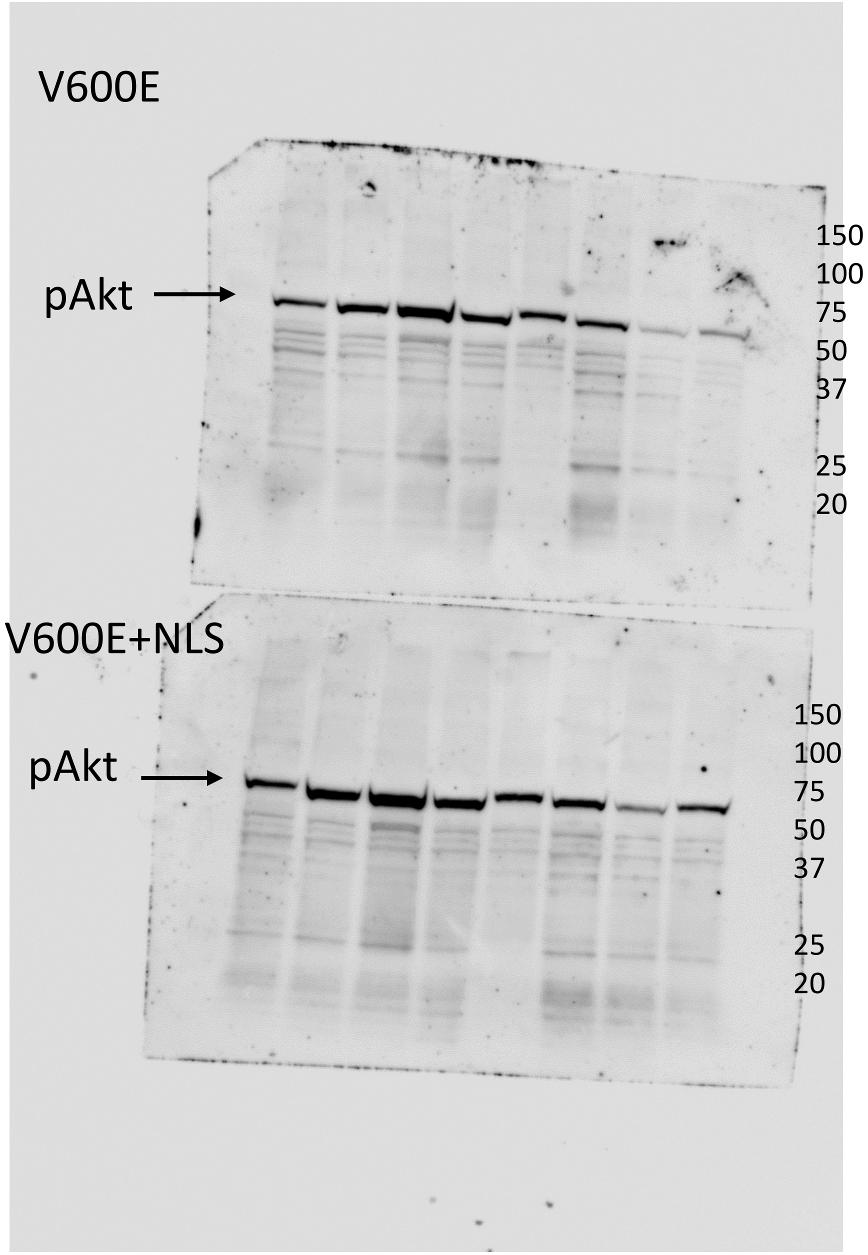

Original blots for Fig. 3D

MEF Cells

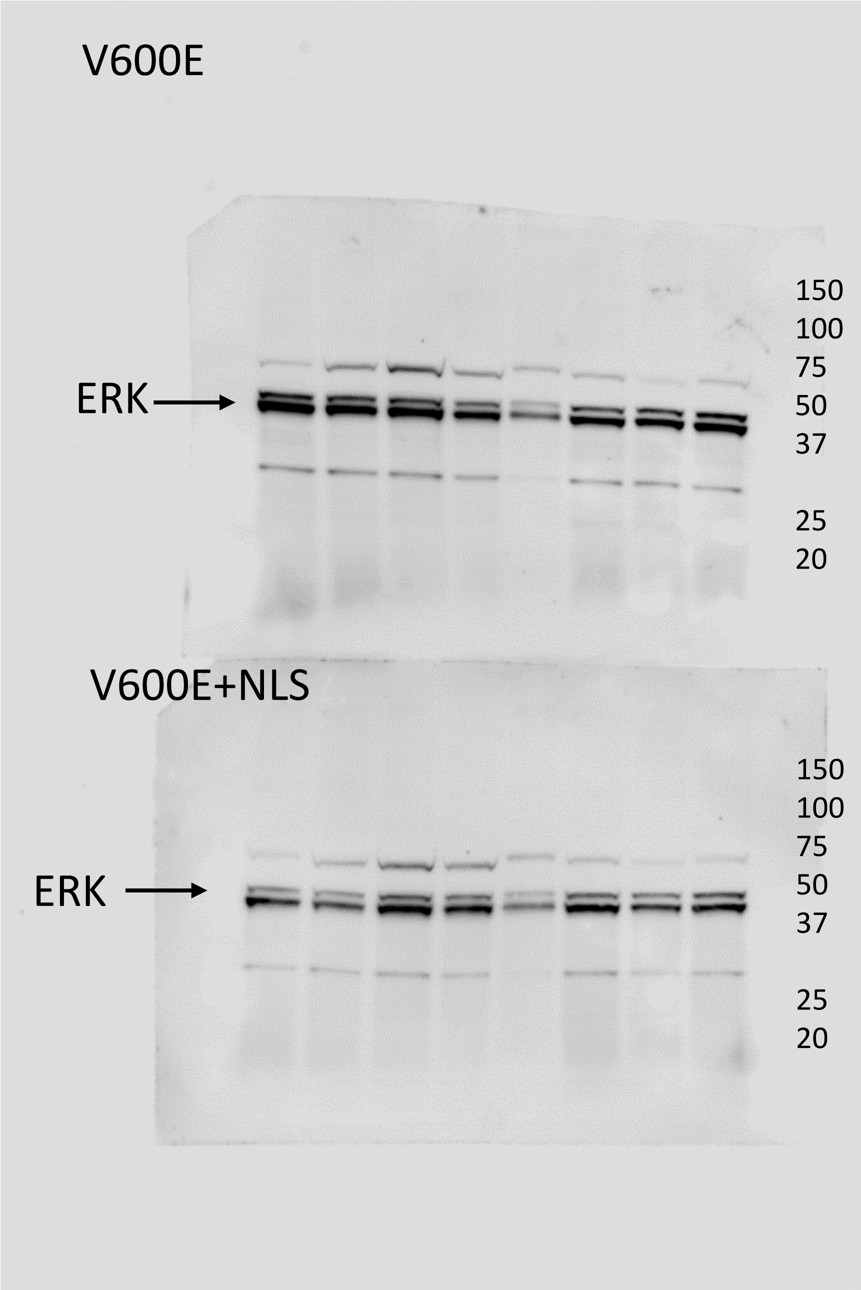

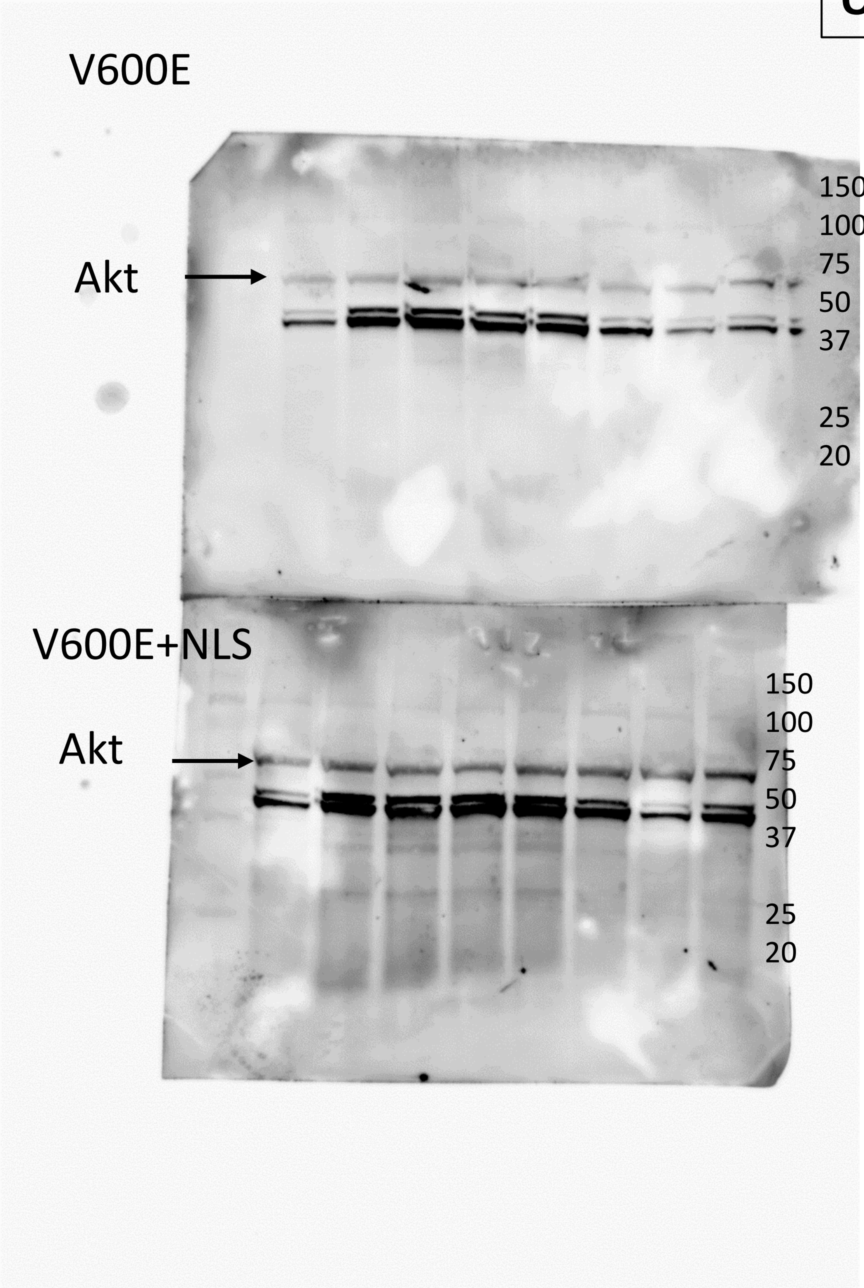

Original blots for Fig. 3D

MEF Cells

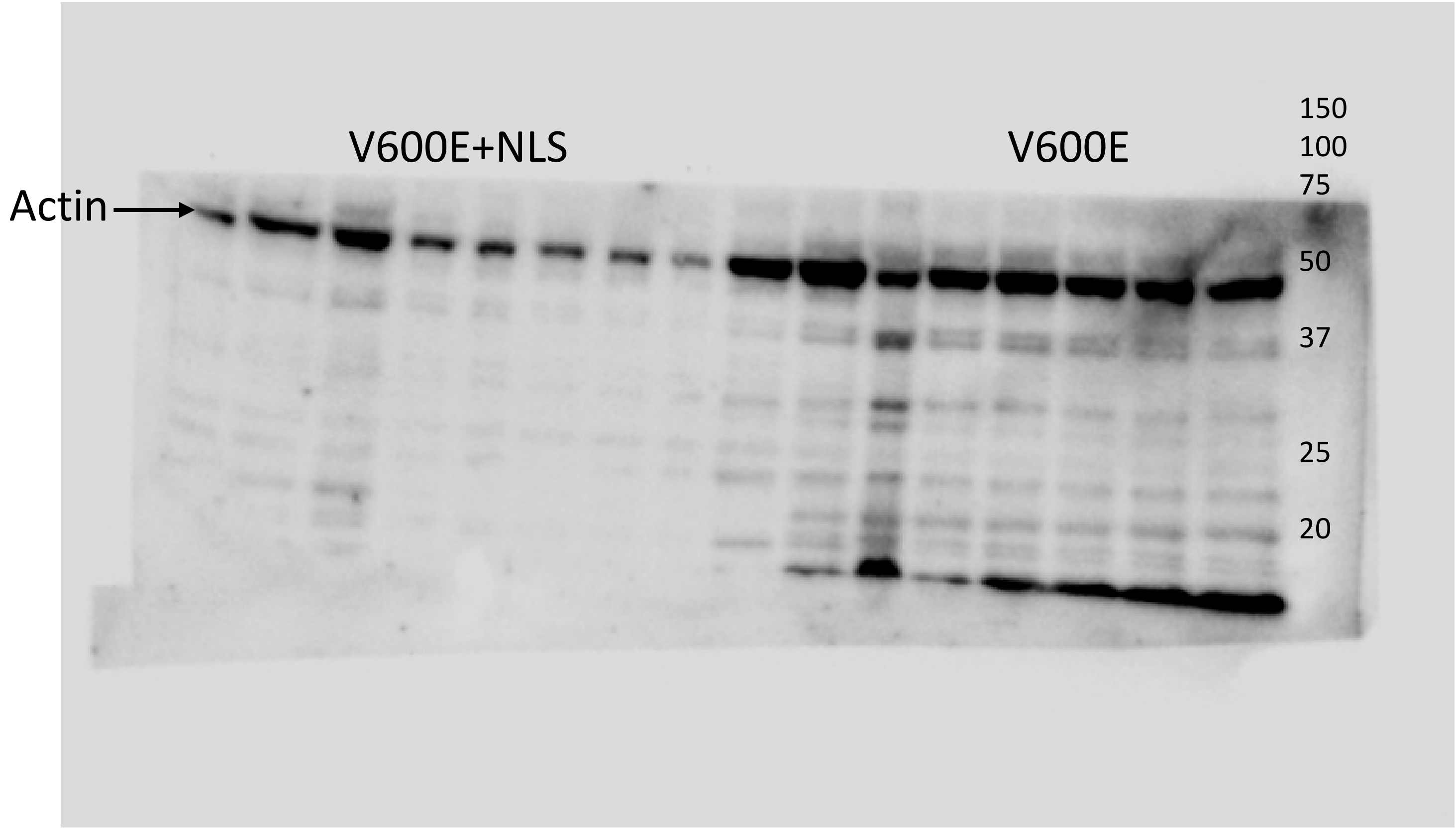

A375R Cells

GAPDH in A375R cells

Original blots for Fig. 1D and 4A

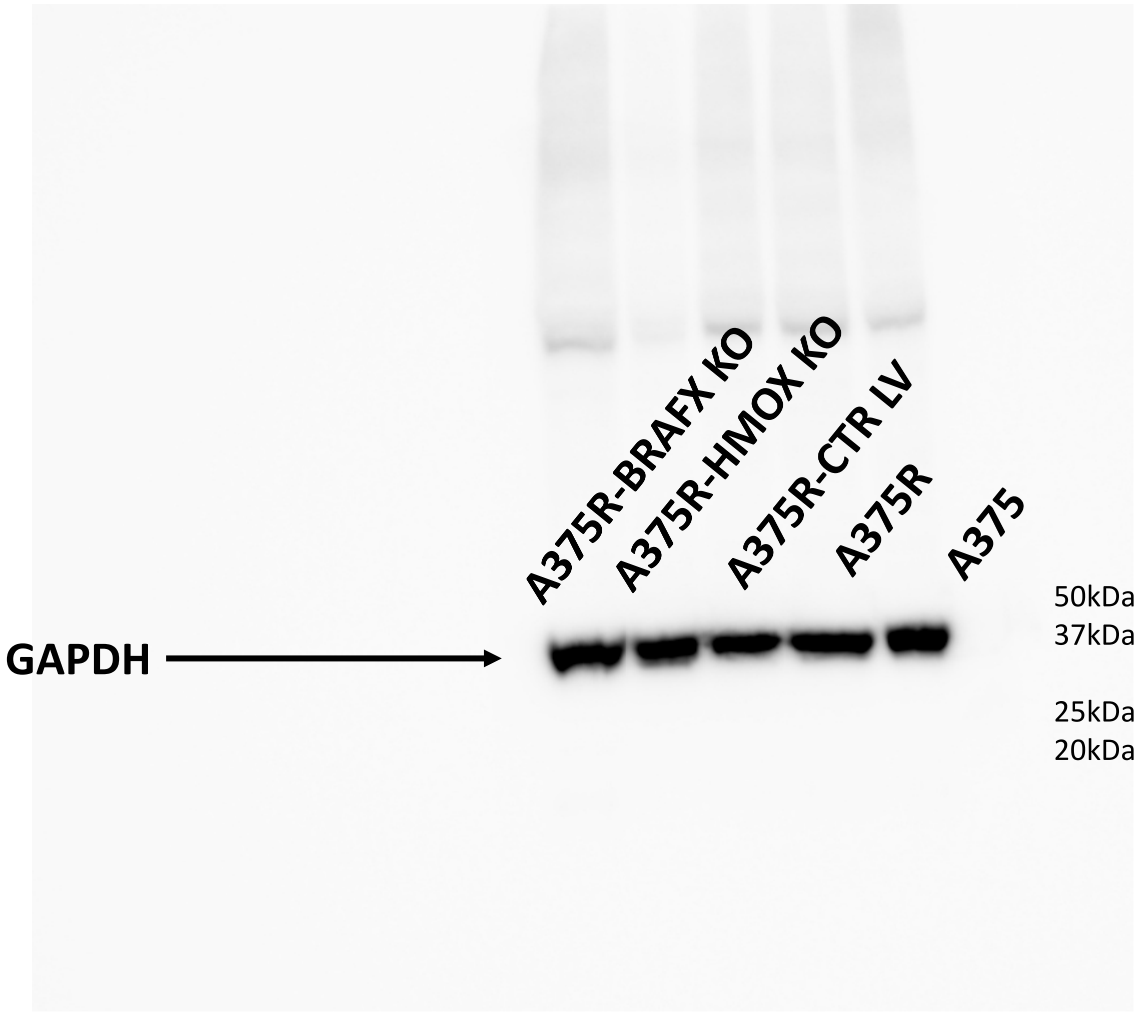

Original blots for Fig. 1D and 4A

A375R Cells

HMOX-1 KO in A375R cells

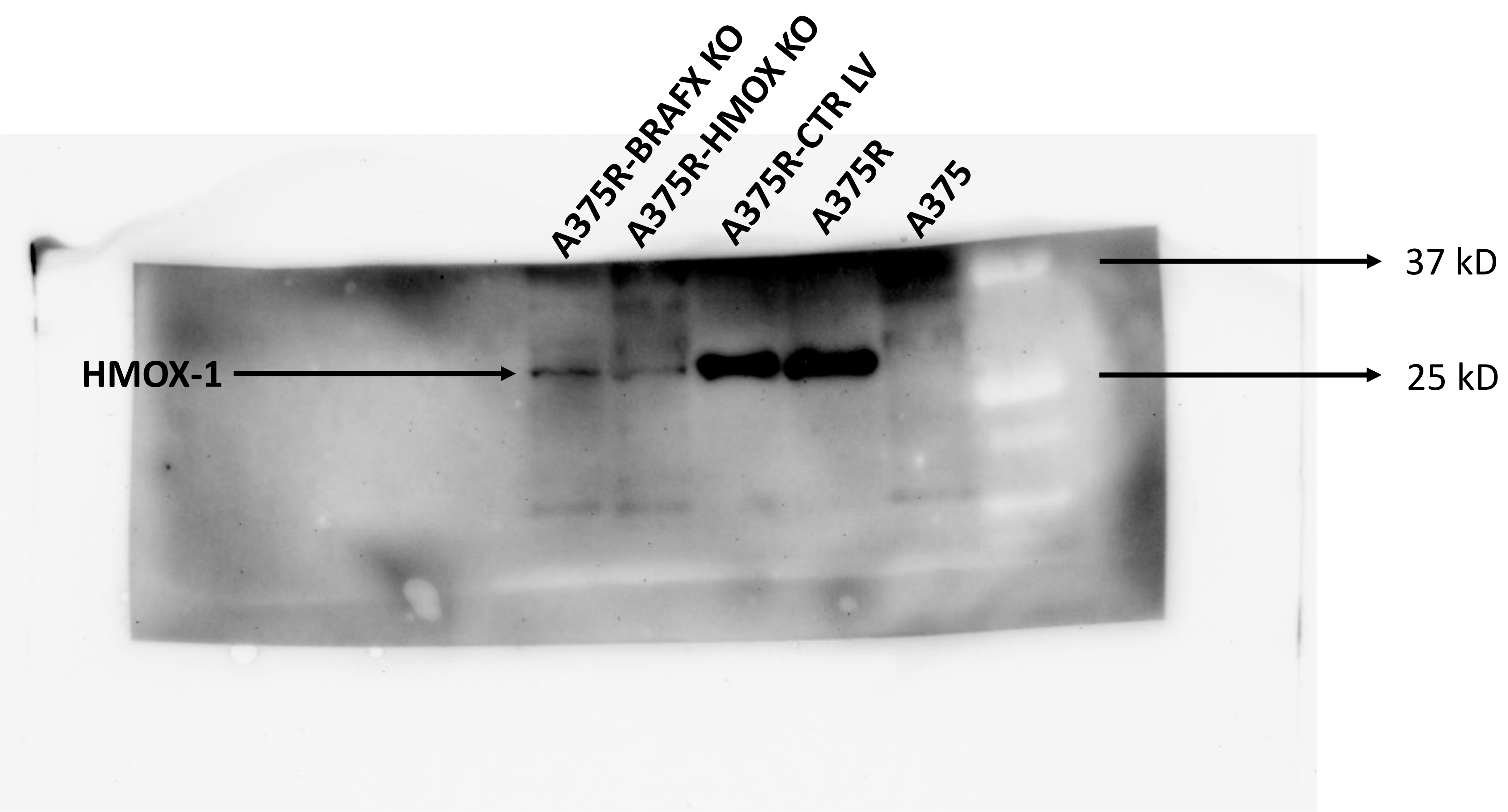

Original blots for Fig. 4A

A375R Cells

BRAF KO in A375R cells

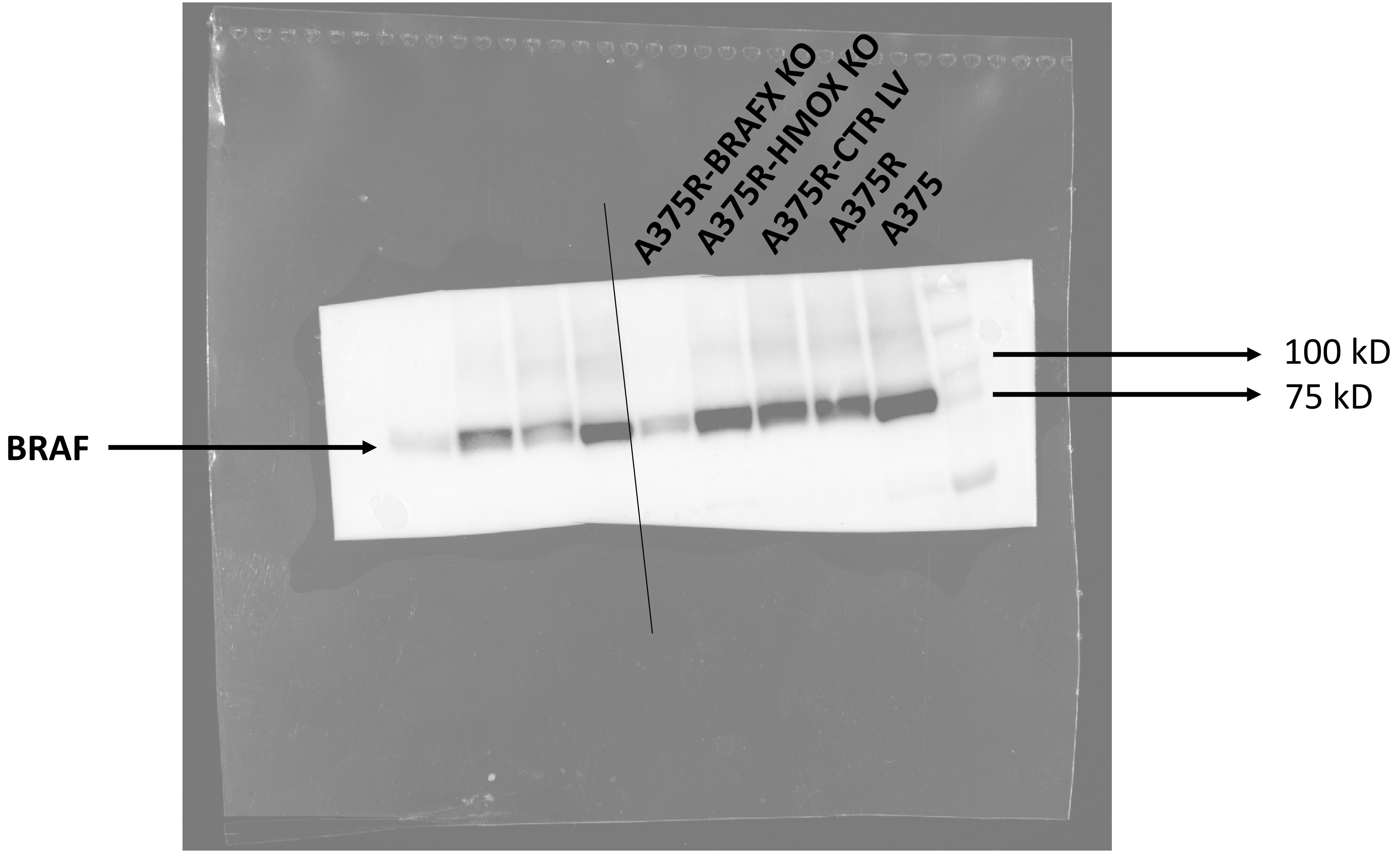

Intensity ratio for Fig. 1D

(D)

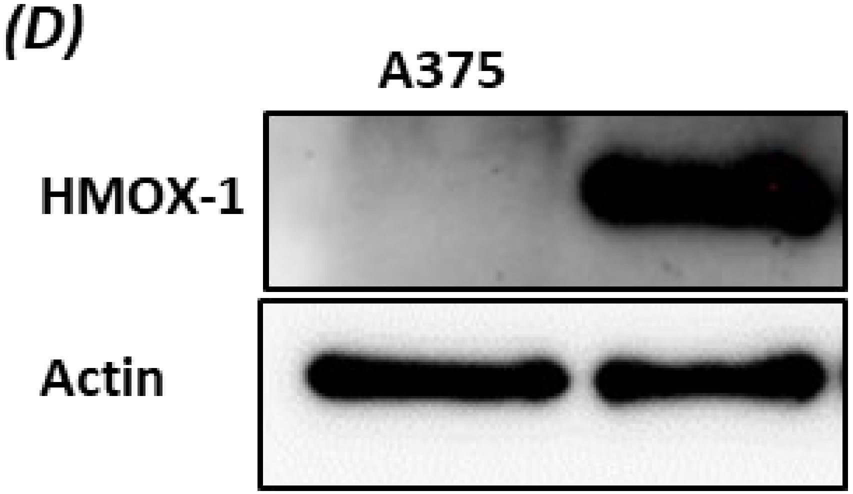

Compared to A375			HMOX1		1	8.3

Intensity ratio for Fig. 3D

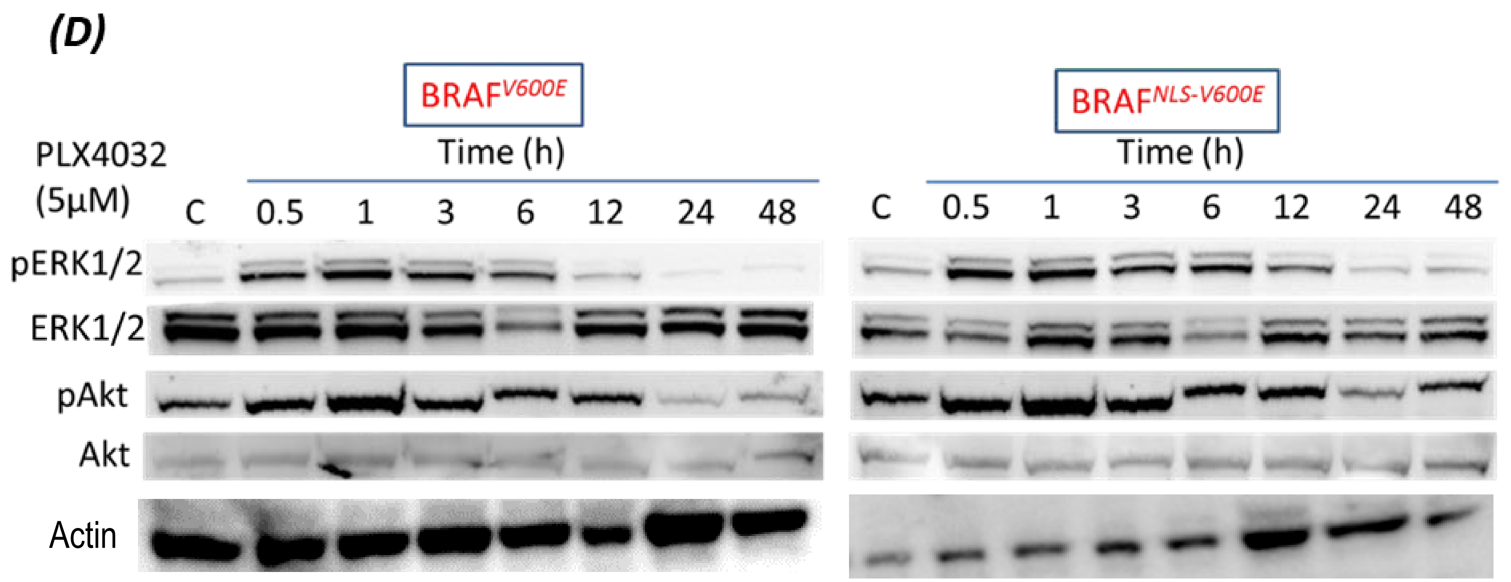

	BRAFV600E)								BRAF(NLSV600E)							
	C	0.5	1	3	6	12	24	48	C	0.5	1	3	6	12	24	48
pERK1/2	1	5.11	6.43	5.22	3.09	1.23	0.32	0.21	1	6.4	6.6	3.3	5.1	2	0.53	0.83
pAKT	1	2.2	4.3	2	1.8	1.9	0.12	0.23	1	3.4	5.3	3.4	2.1	2.6	0.33	1.13
Actin	1	1.2	1.1	1.4	0.9	0.7	1.9	0.9	1	1.2	1.1	1.4	1.3	2.1	1.6	0.9

Intensity ratio for Fig. 4A

(A)

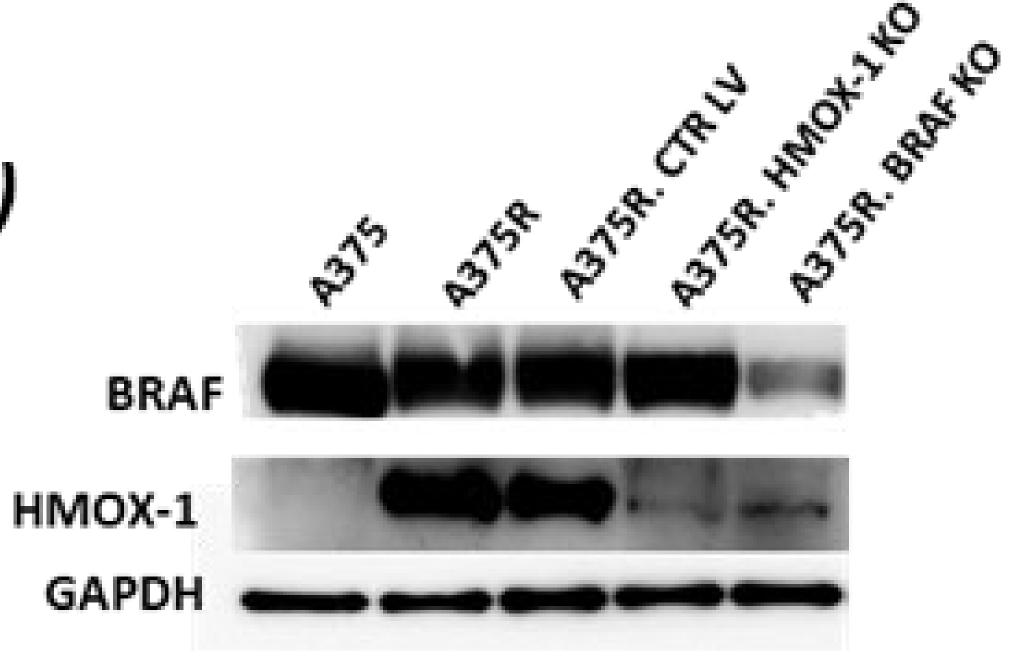

Compared to A375			BRAF	1	0.9	1.02	1.11	0.21	
			HMOX-1	1	37.86	23.89	5.09	7.92	

Original blots for Fig. ***Supplemental Data 5***

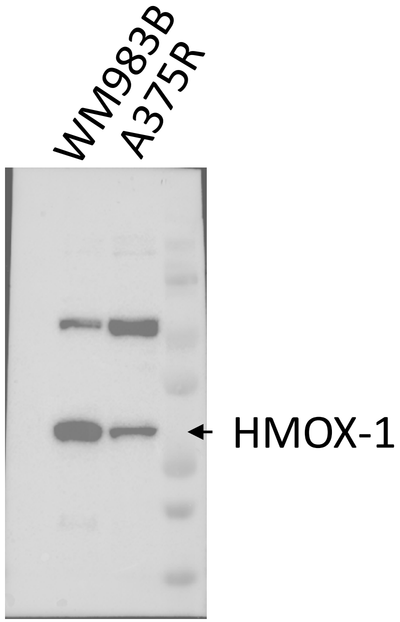

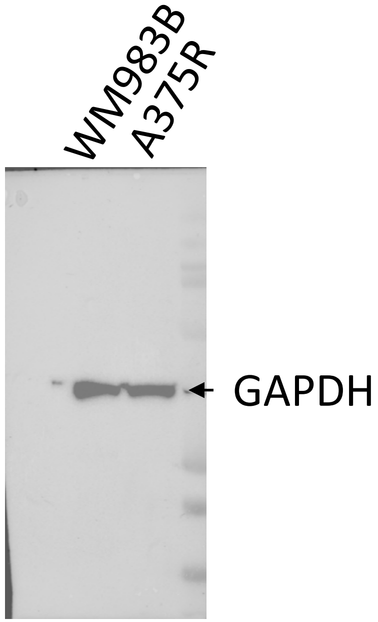

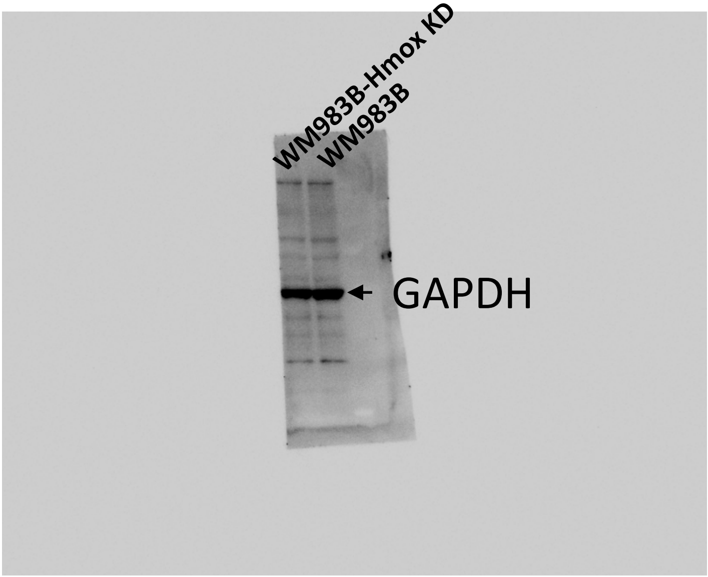

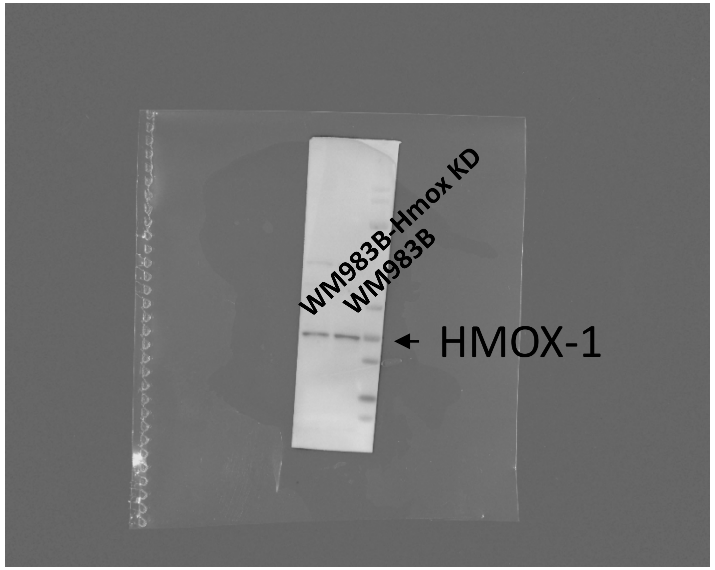

Original blots for Fig. ***Supplemental Data 6***

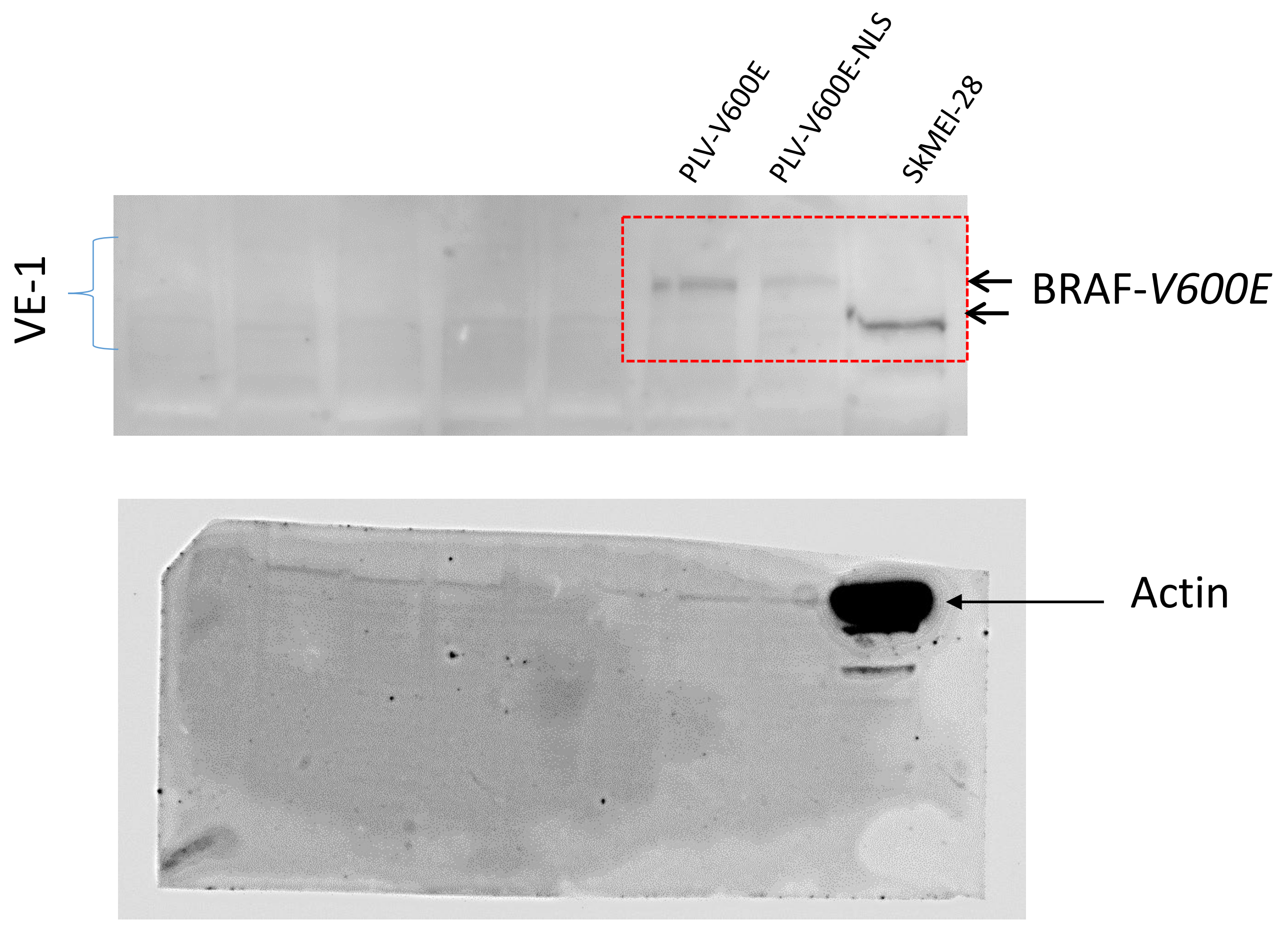

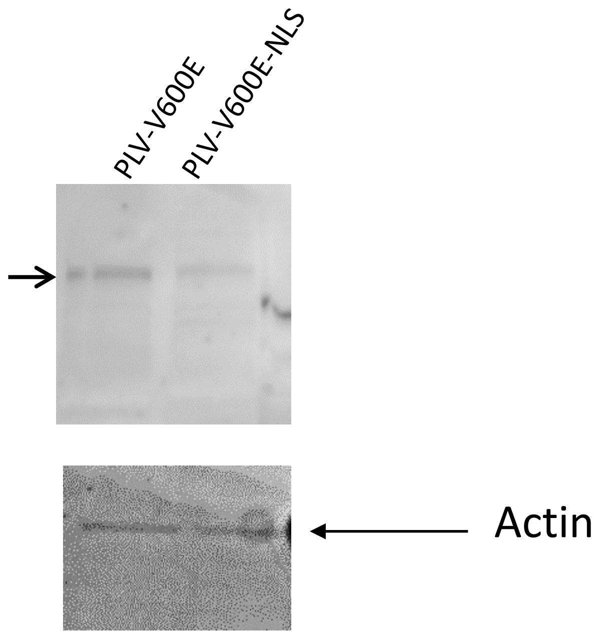

Original blots for Fig. ***Supplemental Data 6***

WT MEF BRAF -/- MEF

BRAF

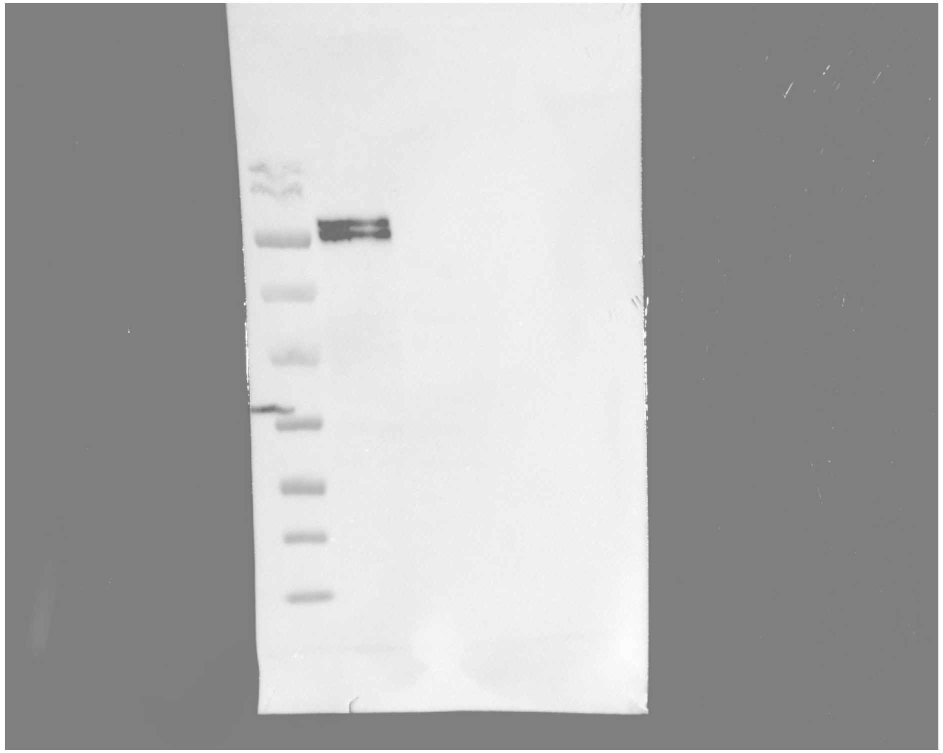

WT MEF BRAF -/- MEF

Actin

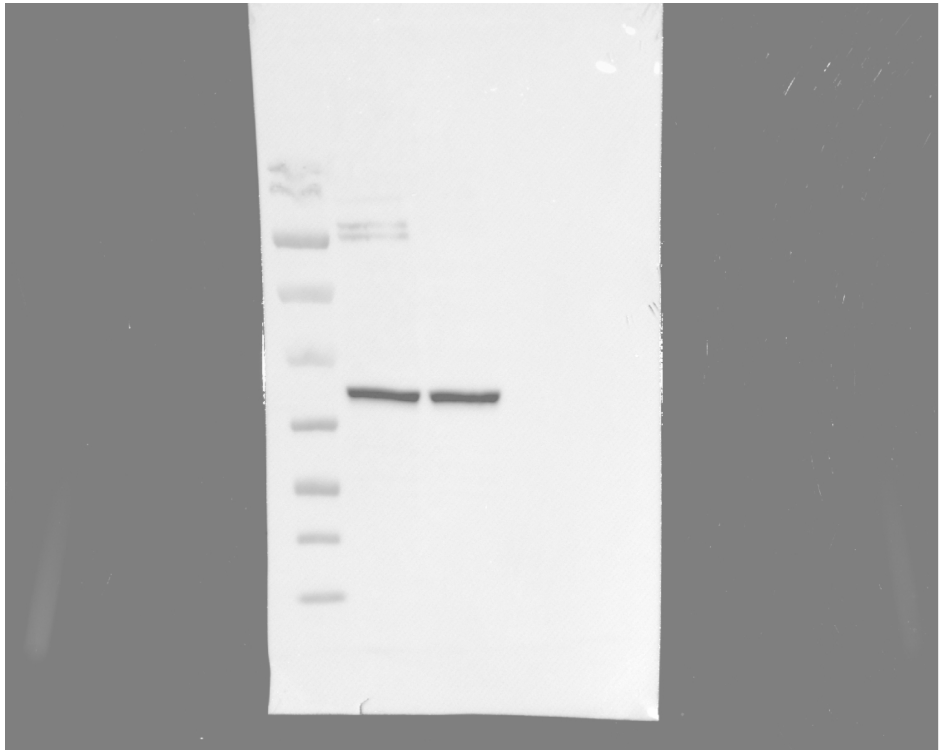

Original blots for Fig. ***Supplemental Data 7***

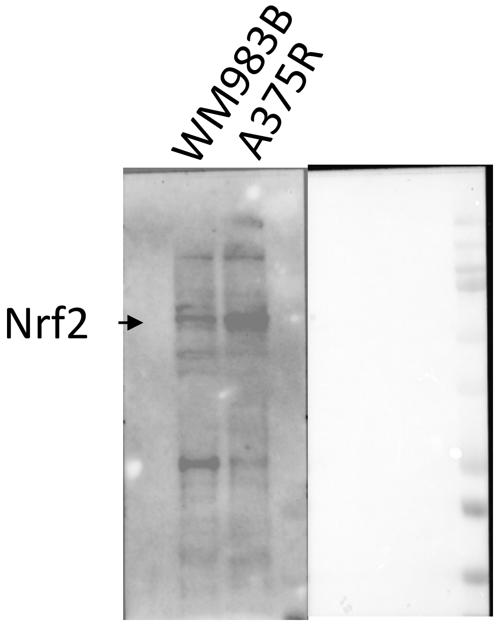

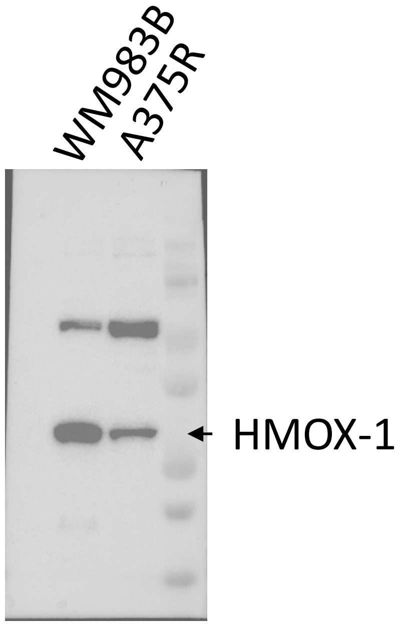

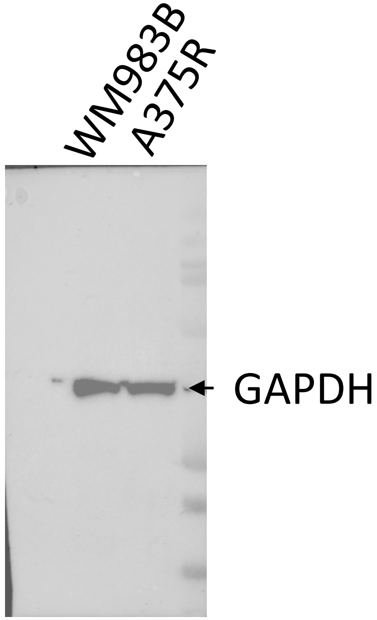

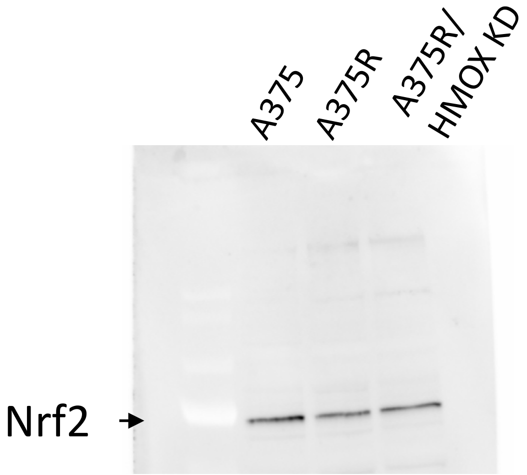

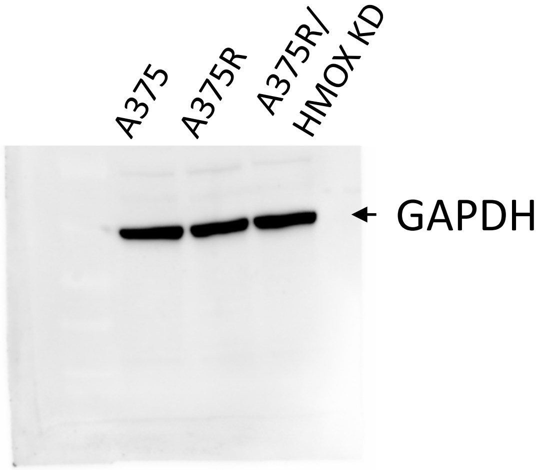

Supplement: Supplementary file 1 [file cancers-14-00311-s001.zip › cancers-1450741-supplementary/cancers-1450741-supplementary WB.pdf]
